# EGF-mediated inhibition of ubiquitin-specific peptidase 24 expression has a crucial role in tumorigenesis

**DOI:** 10.1038/onc.2016.445

**Published:** 2016-12-19

**Authors:** S-A Wang, Y-C Wang, Y-P Chuang, Y-H Huang, W-C Su, W-C Chang, J-J Hung

**Affiliations:** 1 Institute of Bioinformatics and Biosignal Transduction, College of Bioscience and Biotechnology, National Cheng Kung University, Tainan, Taiwan; 2 Department of Biotechnology and Bioindustry Sciences, College of Bioscience and Biotechnology, National Cheng Kung University, Tainan, Taiwan; 3 Institute of Basic Medical Sciences, National Cheng Kung University, Tainan, Taiwan; 4 Department of Pharmacology, National Cheng Kung University, Tainan, Taiwan; 5 Department of Internal Medicine, College of Medicine and Hospital, National Cheng Kung University, Tainan, Taiwan; 6 Graduate Institute of Medical Sciences, College of Medicine, Taipei Medical University, Taipei, Taiwan; 7 The Ph.D. Program for Neural Regenerative Medicine, Taipei Medical University, Taipei, Taiwan; 8 Center for Infectious Disease and Signal Transduction, National Cheng Kung University, Tainan, Taiwan

## Abstract

In this study, several cancer-related proteins (Bax, p300, E2F4 and securin) have been proven to be substrates of ubiquitin-specific peptidase 24 (USP24), and relevance has been shown between USP24 and its substrates in samples from clinical lung cancer patients. Silencing USP24 increases the cancer formation by inhibiting cellular apoptosis and increasing cellular proliferation. Epidermal growth factor (EGF) treatment, and the Kras^G12D^ and EGFR^L858R^ mutations decrease USP24 protein stability via EGF- or CDK1-mediated phosphorylation at Ser1616, Ser2047 and Ser2604. Knockdown of USP24 decreases Bax and p300 levels, and reduces Ku70 acetylation, thereby preventing cancer cell apoptosis. In addition, knockdown of USP24 increases cell cycle progression by enhancing the G1–S transition and metaphase–anaphase transition. The molecular mechanism involves a decrease in the USP24 level, which reduces the expression of E2F4 and its partner TFDP1, and thus increases the G1/S transition. In conclusion, the USP24 level was decreased during the early stage of cancer and the mitotic stage of the cell cycle to regulate its substrates p300, Bax, E2F4 and securin, resulting in decreased cell apoptosis and increased cell cycle progression and, thus, cancer formation.

## Introduction

Ubiquitin-specific peptidases (USPs) belong to a large family of cysteine proteases and are deubiquitinating enzymes that specifically recognize and remove ubiquitin from proteins.^1^ USP disorders have been reported to be involved in many human diseases, such as neurodegenerative disorders, inflammation and cancer progression.^2, 3, 4^ *USP24* was discovered as a genetic variant in Parkinson's disease, where three single-nucleotide polymorphisms (SNPs; rs1165222, rs13312 and rs487230) were associated with Parkinson's disease risk.^5, 6, 7^ However, the functions of USP24 and its detailed mechanisms of action are unknown. Several studies have demonstrated that SNPs in some genes, such as *EGFR* and *p53*, and in microRNAs are related to lung cancer development.^8, 9, 10, 11^ Our recent study showed that *USP24* variants from SNPs and RNA editing products increased the levels of USP24 and MDM2, which regulates Suv39h1 in lung cancer cells, subsequently resulting in an increase in metastatic activities during lung cancer progression.^12^ However, the role of USP24 in cancer formation is unknown.

Decreased cell death and increased cell proliferation are responsible for cancer formation. Therefore, understanding the mechanisms underlying cell apoptosis and cell cycle progression is critical for cancer prevention. Previous studies have shown that the Bax level and mitochondrial localization are important for the induction of apoptosis.^13^ Several proteins, such as Ku70 and p300, have been reported to be involved in apoptosis induction through the control of Bax localization. Ku70 has roles not only in DNA damage but also in Bax-mediated apoptosis. A previous study indicated that Ku70 acetylation increased the mitochondrial localization of Bax, thereby resulting in cell apoptosis.^14, 15^ Our preliminary results from a yeast two-hybrid assay showed that Ku70 interacts with USP24; however, the role of USP24 in Bax-mediated apoptosis is unknown. The regulation of cell cycle progression is an important issue for the control of cell proliferation.^16^ The G1–S transition is a critical stage for cancer formation. E2F is a group of eukaryotic transcription factors; three members of the group (E2F1, E2F2 and E2F3) are activators, whereas the other members (E2F3b and E2F4-8) act as suppressors.^17^ Previous studies indicate that E2F4 is involved in the regulation of cell cycle progression through inhibiting the transcription activity of E2F1.^18^ Most of these proteins are involved in cell cycle regulation and DNA synthesis in mammalian cells through the formation of a homodimer or heterodimer with TFDP1.^19^ Securin has been reported to be involved in the control of the metaphase–anaphase transition. In addition, securin interacts with separase to inhibit its activity. During metaphase, securin is ubiquitinated by the APC/C E3 ligase to degrade securin, resulting in the release of separase from securin, the degradation of cohesion and subsequent entry into anaphase. Therefore, securin downregulation is important for the metaphase–anaphase transition.^20^ Although the role of APC/C as an E3 ligase that regulates the securin level has been well studied, the role of deubiquitinating proteins in the control of the securin level is unknown and is addressed in this study.

Herein, we found that USP24 was downregulated by phosphorylation during the early stage of cancer and the mitotic stage of cell cycle progression to inhibit apoptosis and increase cell cycle progression. Several proteins have been identified as the substrates of USP24; these proteins regulate cell apoptosis and cell cycle progression, which are important for cancer formation.

## Results

### EGF-inhibited USP24 expression induces cancer formation

**Figure 1:**
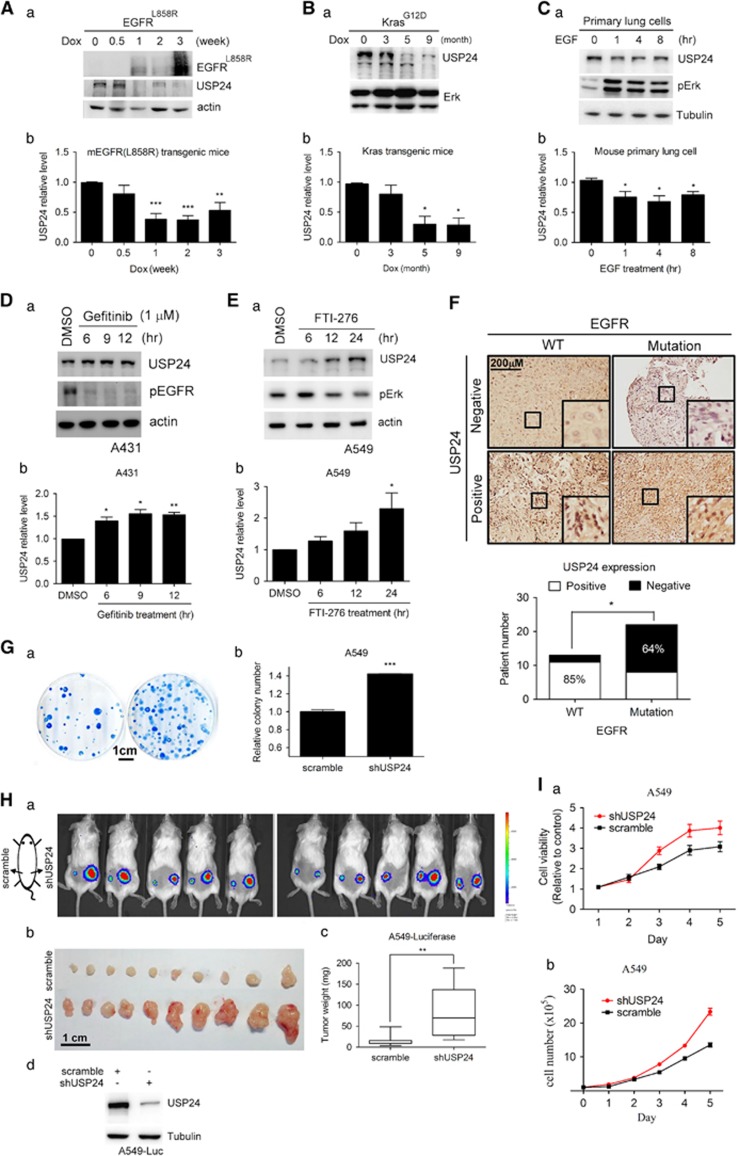
EGF-inhibited USP24 expression induces cancer formation. (**A–C**) Samples from EGFR^L858R^- and Kras^G12D^-induced lung cancer mice or primary lung cancer cells were collected to study the EGFR^L858R^, USP24, ERK1/2, p-ERK, actin and tubulin levels by western blotting with antibodies against the indicated proteins (a). The USP24 level was quantified in three independent experiments (b). (**D**, **E**) Samples were collected from gefitinib-treated A431 and FTI-276-treated A549 cells, and the USP24, p-EGFR, p-ERK, actin and tubulin levels were determined by western blotting with antibodies against the indicated proteins (a). The USP24 level was quantified after three independent experiments (b). (**F**) The USP24 level was examined in the specimens from lung cancer patients with or without EGFR mutations using immunohistochemistry with an anti-USP24 antibody. (**G**) Cell proliferation was studied in A549 cells with or without USP24 knockdown using the colony assay (a). The colony numbers were counted (b). (**H**) A549-luciferase-expressing cells with or without USP24 knockdown were injected into severe combined immunodeficiency mice. After 3 weeks, tumors in the mice were detected by *in vivo* imaging system assay (a). The mice were killed, and tumor size (b) and tumor weight (c) were measured, and USP24 level shown here as an internal control (d). (**I**) The cell viability (a) and cell numbers (b) were evaluated in A549 cells with or without USP24 knockdown. *P<0.1; **P<0.05; ***P<0.01, *t-*test.

To study the role of USP24 during tumorigenesis, we assessed USP24 levels in two doxycycline-induced lung cancer transgenic mouse models (Kras^G12D^ and EGFR^L858R^; Figures 1A and B; Supplementary Figures 1A and B). Adenocarcinomas were induced after doxycycline treatment of the Kras^G12D^ and EGFR^L858R^ transgenic mice (Supplementary Figures 1A and B). The USP24 level was decreased under doxycycline treatment in the induced EGFR^L858R^ and Kras^G12D^ mice (Figures 1A and B). USP24 expression was also decreased in epidermal growth factor (EGF)-treated primary lung cells (Figure 1C). Gefitinib and FTI-276, which inhibited the EGFR and Kras activities^21^ in the A431 and A549 cell lines, respectively, increased USP24 expression (Figures 1D and E). Next, we examined the USP24 mRNA level and found that there was no difference, indicating that the USP24 reduction observed here was not due to a decrease in its transcriptional activity (Supplementary Figures 1C–E). We investigated the relevance between the USP24 level and the EGFR mutation in clinical lung cancer specimens and found that patients with the EGFR mutation were more highly correlated with reduced USP24 expression (Figure 1F). Taken together, these data revealed that Kras^G12D^- or EGFR^L858R^-mediated pathways negatively regulated USP24 expression, which might trigger lung cancer formation. Knockdown of USP24 in A549 cells increased colony formation (Figure 1G). The injection of luciferase-expressing A549 cells with USP24 knockdown into severe combined immunodeficiency mice increased the tumor size and weight (Figure 1H). Finally, USP24 knockdown increased the cell numbers and cell viability in A549 and lung primary cells, suggesting that USP24 negatively regulated lung cancer formation (Figure 1I; Supplementary Figures 1F–I).

### USP24 induces apoptosis by stabilizing p300 and Bax

**Figure 2:**
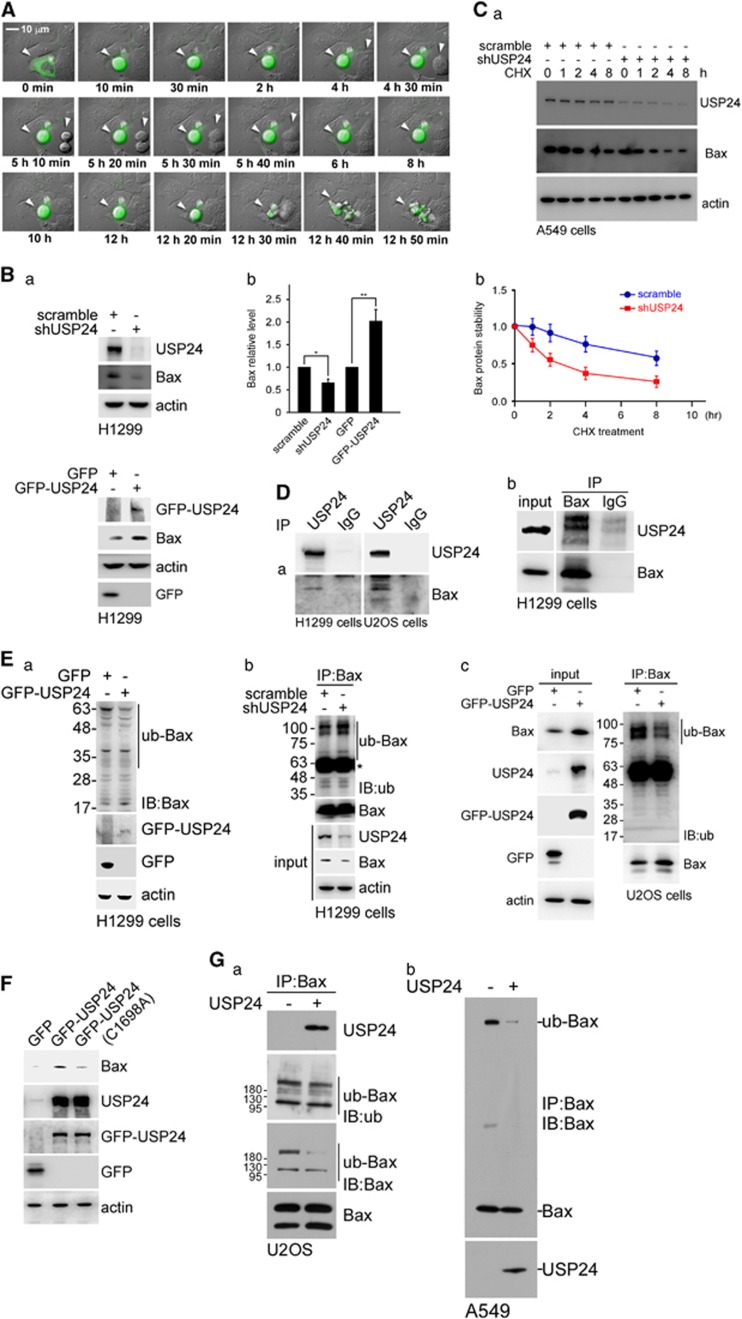
USP24 induces apoptosis by stabilizing Bax. (**A**) GFP-USP24 was transfected into H1299 cells and the cellular morphology was assessed using time-lapse fluorescence microscopy. Indicated time sequence is the record time of time lapse after transfection 16 h. (**B**) Samples were collected from GFP-USP24 overexpressing and USP24 knockdown H1299 cells, and the Bax USP24, EGFP-USP24, GFP and actin levels were examined by western blotting with antibodies against the indicated proteins (a). The USP24 level was quantified for the statistical analysis after three independent experiments (b). (**C**) USP24 was knockdown in A549 cells, and samples were collected after cycloheximide treatment. The Bax, actin and USP24 levels were studied by western blotting with antibodies against the indicated proteins (a). The Bax level was quantified for the statistical analysis after three independent experiments (b). (**D**) Samples were collected from H1299 cells for immunoprecipitation with anti-USP24 (a) and anti-Bax antibodies (b). Immunoprecipitated (IP) samples were used to study the USP24 and Bax levels by western blotting with anti-USP24 and anti-Bax antibodies. (**E**) GFP-USP24 was expressed in H1299 cells, and then samples were collected for western blotting with antibodies against indicated proteins (a). USP24 was knockdown in H1299 cells, and then cells were collected for immunoprecipitation assay with anti-Bax antibodies. IP samples were analyzed by western blotting with antibodies against the indicated proteins (b). Samples were collected from U2OS cells expressing GFP-USP24 for immunoprecipitation with an anti-Bax antibody. IP samples were used to assess the ubiquitin-Bax and Bax by western blotting with anti-ubiquitin and anti-Bax antibodies (c). (**F**) Samples were collected from U2OS cells with GFP-USP24 or GFP-USP24(C1698A) expression to determine the Bax, USP24, GFP-USP24, GFP and actin levels by western blotting with antibodies against the indicated proteins. (**G**) Samples were collected from U2OS (a) or A549 (b) cells for immunoprecipitation with an anti-Bax antibody. IP samples were used to perform the *in vitro* deubiquitination assay with or without purified USP24 protein (50 μg/ml) and then determine the ubiquitin, Bax and USP24 levels by western blotting with antibodies against the indicated proteins.

**Figure 3:**
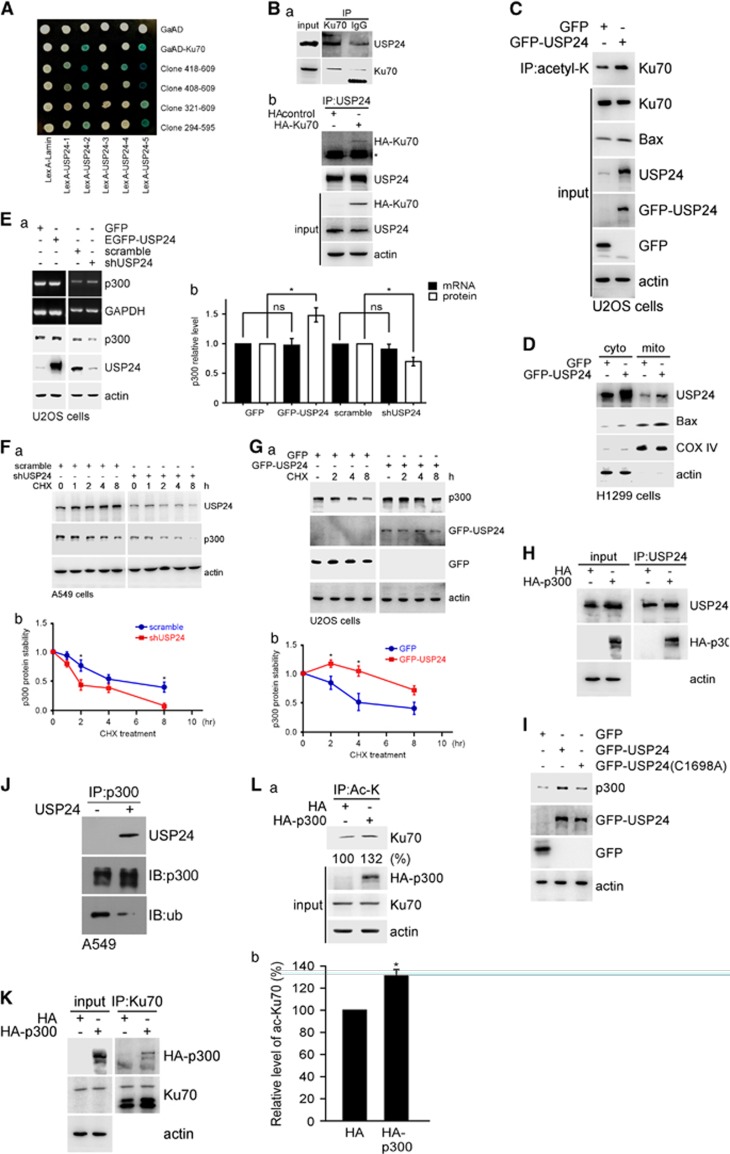
USP24 interacted with p300 involves in the acetylation of Ku70 to regulate the mitochondria localization of Bax. (**A**) The interaction between Ku70 including its indicated truncated domains and USP24 including the indicated truncated domains were evaluated in the yeast two-hybrid assay. (**B**) Samples were collected from A549 cells for immunoprecipitation with anti-Ku70 antibody. Immunoprecipitated (IP) samples were used to study the USP24 and Ku70 levels by western blotting with anti-USP24 and anti-Ku70 antibodies (a). Samples were collected from A549 cells with or without HA-Ku70 expression for immunoprecipitation with anti-USP24 antibody. IP samples were used to study the USP24 and HA-Ku70 levels by western blotting with antibodies against the indicated proteins (b). (**C**) Samples were collected from U2OS cells with GFP-USP24 overexpression for immunoprecipitation with anti-acetylated lysine antibodies. IP samples were used to investigate the acetylated Ku70 level by immunoblotting with an anti-Ku70 antibody. The Ku70, Bax, USP24, GFP-USP24, GFP and actin levels were examined by western blotting with antibodies against the indicated proteins. (**D**) Cellular extracts from H1299 cells with GFP-USP24 overexpression were divided into the cytoplasmic and mitochondrial fractions. The samples were used to study the USP24, Bax, COX IV and actin levels by western blotting with antibodies against the indicated proteins. (**E**) Samples were collected from U2OS cells with GFP-USP24 expression or USP24 knockdown to evaluate the p300, USP24 and actin levels by western blotting. The p300 and GAPDH mRNA levels were evaluated by reverse transcription–PCR. Knocked down in A549 cells (**F**) or GFP-USP24 was overexpressed in U2OS cells (**G**), and samples were collected after cycloheximide treatment. The p300, actin, GFP-USP24 and GFP levels were studied by western blotting with antibodies against the indicated proteins (a). The p300 level was quantified for the statistical analysis after three independent experiments (b). (**H**) Samples were collected from U2OS cells overexpressing HA-p300 for the immunoprecipitation assay with a USP24 antibody. IP samples were used to study the USP24, HA-p300 and actin levels. (**I**) Samples were collected from cells overexpressing GFP-USP24 or GFP-USP24(C1698A) to study the p300, GFP-USP24, GFP and actin levels by western blotting with antibodies against the indicated proteins. (**J**) Samples were collected from A549 cells for immunoprecipitation with an anti-p300 antibody. IP samples were used to perform the *in vitro* deubiquitination assay with recombinant human USP24 (50 μg/ml) and then evaluate the USP24, p300 and ubiquitinated p300 levels with antibodies against the indicated proteins. (**K**) Samples were collected from U2OS cells overexpressing HA-p300 for the immunoprecipitation assay with a Ku70 antibody. IP samples were used to study the Ku70, HA-p300 and actin levels. (**L**) Samples were collected from U2OS cells overexpressing HA-p300 for the immunoprecipitation assay with an acetyl-lysine antibody. The IP samples were used to study the acetylated Ku70 levels by western blotting with an anti-Ku70 antibody (a). The acetylated Ku70 level was quantified for the statistical analysis after three independent experiments (b).

To investigate the molecular mechanism by which USP24 inhibited lung cancer formation, H1299 cells with GFP-USP24 or GFP expression were recorded using time-lapse microscopy (Figure 2A; Supplementary Figure 2A). GFP-USP24 expression, but not GFP, led to rounded cells and, finally, cell death. As most of the previous studies about USP24 were performed in U2OS cells, herein we not only use lung cancer cell lines but also use U2OS to study the role of USP24 in tumorigenesis. Knockdown of USP24 decreased the Bax and caspase-3 levels; conversely, these protein levels were increased in GFP-USP24-expressing cells, although no alterations were observed in the Bax mRNA level (Figure 2B; Supplementary Figure 2B). USP24 knockdown decreases Bax protein stability in A549 and U2OS cells (Figure 2C; Supplementary Figure 2C). MG132 treatment rescued the Bax level, indicating that USP24 increased the Bax level by enhancing protein stability, thereby contributing to cell apoptosis (Supplementary Figure 2D). Knockdown of USP24 increased the total ubiquitination signal, implying that USP24 is a deubiquitinating enzyme involved in controlling protein stability (Supplementary Figure 2E). Next, we found that USP24 interacted with Bax (Figure 2D). GFP-USP24 overexpression decreased the ubiquitinated Bax signal, and this effect was reversed by USP24 knockdown (Figure 2E). However, mutation of GFP-USP24 at Cys1698 (GFP-USP24(C1698A)) abolished the effect of GFP-USP24-wt on the Bax level, indicating that USP24 enzymatic activity was essential for Bax stabilization (Figure 2F; Supplementary Figures 2F–H). The *in vitro* deubiquitinating enzymatic assay using the purified USP24 protein indicated that Bax ubiquitination was decreased in the presence of USP24, suggesting that Bax was a substrate of USP24 (Figure 2G). Previous studies indicated that Ku70 was involved in Bax mitochondrial translocation.^15^ Herein, we used a yeast two-hybrid assay to study the interaction between Ku70 and USP24 (Figure 3A; Supplementary Figure 2I). The data revealed that USP24 interacted with Ku70 (Figure 3B). Overexpression or knockdown of USP24 did not affect the Ku70 level but increased or decreased Ku70 acetylation, respectively (Figure 3C; Supplementary Figure 2J). The localization of Bax in cells expressing GFP-USP24 indicated that USP24 expression increased Bax's mitochondrial localization, implying that USP24 induced cell apoptosis (Figure 3D). Overexpression of GFP-USP24 increased the p300 level but did not affect its mRNA level. Similarly, knockdown of USP24 decreased the p300 level but did not affect its mRNA level (Figure 3E). Thus, GFP-USP24 increased p300 protein stability, whereas knockdown USP24 decreased its stability (Figures 3F and G; Supplementary Figure 2K) and USP24 interacted with p300 (Figure 3H). However, GFP-USP24-C1698A abolished the effect of GFP-USP24-wt on the p300 level, indicating that USP24 enzymatic activity was essential for p300 stabilization (Figure 3I). The *in vitro* enzymatic assay using the purified USP24 protein demonstrated that USP24 decreased the p300 ubiquitination signal (Figure 3J). In addition, Ku70 interacted with HA-p300 (Figure 3K) and HA-p300 overexpression increased Ku70 acetylation (Figure 3L). Based on these data, Bax and p300 were substrates of USP24. USP24 increased the Bax levels and Ku70 acetylation-mediated Bax mitochondrial transportation, resulting in cell apoptosis (Figure 3).

### USP24 inhibits the G1–S transition by increasing the E2F4 level

**Figure 4:**
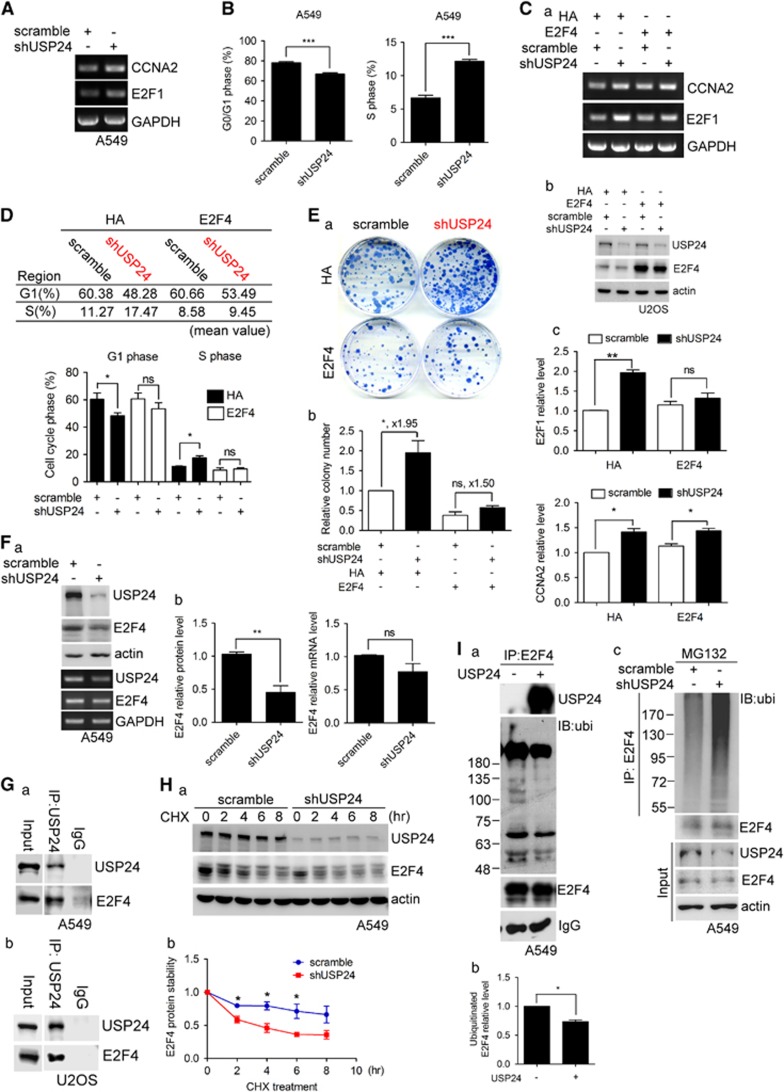
USP24 inhibits the G1–S transition by increasing the E2F4 level. (**A**) Total RNA was isolated from USP24-silenced A549 cells to investigate the CCNA2, E2F1 and GAPDH mRNA levels by reverse transcription–PCR (RT–PCR). (**B**) A549 cells with USP24 knockdown were collected for flow cytometry. After three independent experiments, the ratio of G0/G1 and S-phase was quantified. (**C**) The mRNA was isolated from U2OS cells with USP24 knockdown or E2F4 overexpression to investigate CCNA2, E2F1 and GAPDH mRNA expression with RT–PCR (a). The proteins was isolated from U2OS cells with USP24 knockdown or E2F4 overexpression to investigate USP24, E2F4 and actin protein expression by western blotting (b). The CCNA2, E2F1 and GAPDH mRNA levels were quantified after three independent experiments (c). (**D**) Cells with USP24 knockdown or with E2F4 overexpression were collected for the flow cytometry assay. The G1 and S-phase ratio was quantified after three independent experiments. (**E**) Cells with USP24 knockdown or with E2F4 overexpression were collected for the colony assay (a). The colony number was calculated after three independent experiments (b). (**F**) Total mRNA and proteins were collected from A549 cells with USP24 knockdown to study the USP24, E2F4 and GAPDH mRNA levels by RT–PCR and the USP24, E2F4 and actin protein levels by western blotting. The protein and mRNA levels were quantified after three independent experiments. (**G**) A549 (a) and U2OS (b) were collected for the immunoprecipitation assay with an anti-USP24 antibody. Immunoprecipitated (IP) samples were used for western blotting with antibodies against the indicated proteins. (**H**) A549 cells with USP24 kockdown were collected after cycloheximide treatment. The USP24, E2F4 and actin levels were studied by western blotting with antibodies against the indicated proteins. The E2F4 level was quantified for the statistical analysis after three independent experiments. (**I**) Samples were collected from A549 cells for immunoprecipitation with an anti-E2F4 antibody. IP samples were used to perform the *in vitro* deubiquitination assay with recombinant human USP24 (50 μg/ml) and then evaluate the USP24, E2F4 and ubiquitinated E2F4 levels with antibodies against the indicated proteins (a). The ubiquitinated E2F4 level was quantified for the statistical analysis after three independent experiments (b). A549 cells with USP24 knockdown were collected for IP with an anti-E2F4 antibody. The IP samples were used for western blotting with antibodies against the indicated proteins (c).

**Figure 5:**
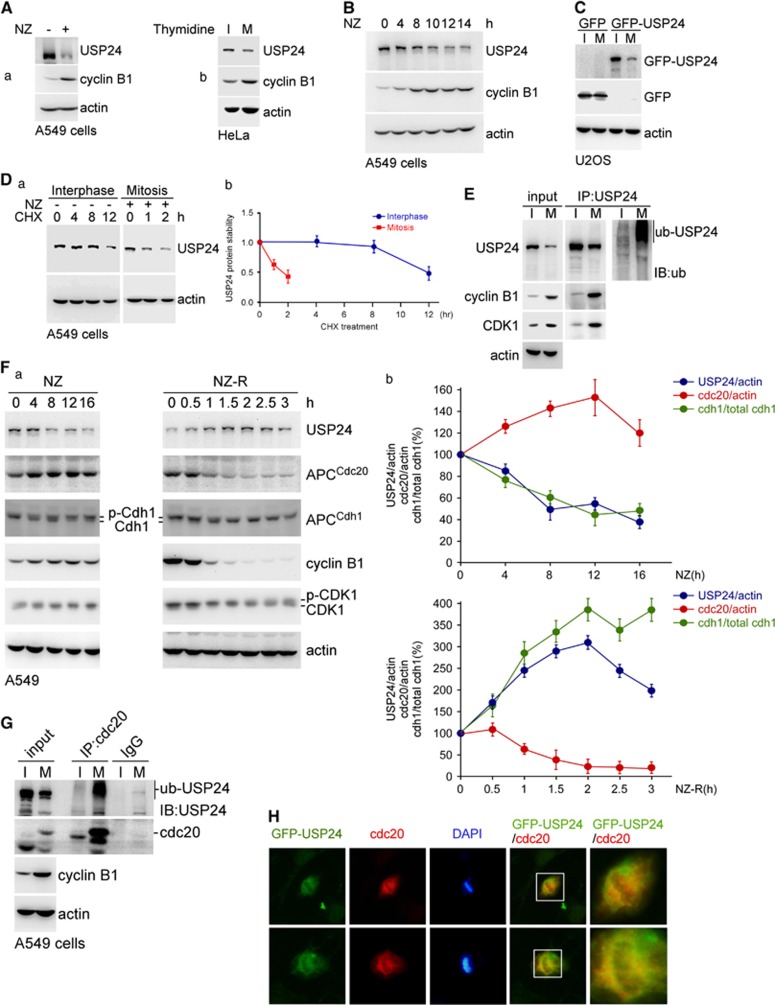
CDK1 interacted with USP24 involves in the decrease in USP24 during mitosis. (**A**) A549 cells treated with nocodazole (a) or thymidine (b) were collected for western blotting with antibodies against the indicated proteins. (**B**) A549 cells treated with nocodazole were collected at the indicated time points for western blotting with antibodies against the indicated proteins. (**C**) U2OS cells with GFP or GFP-USP24 overexpression were collected for western blotting with an anti-GFP antibody. (**D**) A549 cells were treated with nocodazole and cycloheximide, and samples were collected for western blotting with anti-USP24 and anti-actin antibodies (a). The USP24 level was quantified after three independent experiments (b). (**E**) A549 cells with or without nocodazole treatment were collected for immunoprecipitation with an anti-USP24 antibody. Immunoprecipitated (IP) samples were used for western blotting with antibodies against the indicated proteins. (**F**) A549 cells treated with nocodazole (NZ) or nocodazole release (NZ-R) for the indicated time points were collected for western blotting with antibodies against the indicated proteins (a). The protein levels were quantified after three independent experiments (b). (**G**) A549 cells treated with nocodazole were collected for the immunoprecipitation assay with anti-CDC20. The IP samples were used for western blotting with antibodies against the indicated proteins. (**H**) A549 cells with GFP-USP24 overexpression were used for the immunofluorescence assay with antibodies against the indicated proteins.

**Figure 6:**
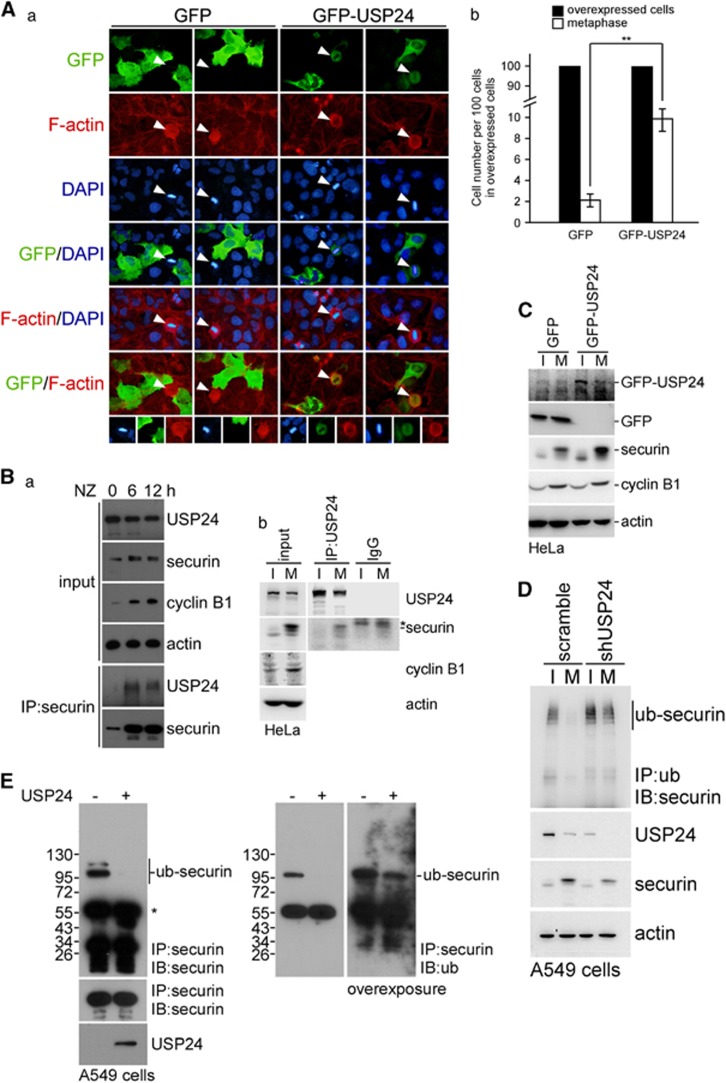
USP24 downregulation in mitosis is involved in the transition of metaphase/anaphase. (**A**) A549 cells with GFP or GFP-USP24 overexpression were used for the immunofluorescence assay with an anti-GFP antibody and stained with DAPI (a). Cells with GFP or GFP-USP24 expression were quantified, and metaphase cells expressing GFP or GFP-USP24 were also quantified (b). (**B**) Cells treated with nocodazole were collected for the immunoprecipitation assay with anti-securin. The Immunoprecipitated (IP) samples were used for western blotting with antibodies against the indicated proteins (a). HeLa cells with or without nocodazole treatment were collected for immunoprecipitation with an anti-USP24 antibody. IP samples were used for western blotting with antibodies against the indicated proteins (b). (**C**) HeLa cells overexpressing GFP-USP24 were used for western blotting with antibodies against the indicated proteins. (**D**) A549 cells with USP24 knockdown treated with nocodazole were collected for the immunoprecipitation assay with an anti-ubiquitin antibody. IP samples were used for western blotting with antibodies against the indicated proteins. (**E**) A549 cells were collected for the immunoprecipitation assay with anti-securin. IP samples were used for the *in vitro* deubiquitination assay with the purified USP24 protein (50 μg/ml). Then, the samples were used for western blotting with antibodies against the indicated proteins.

Next, we investigated the role of USP24 in cell cycle progression (Figures 4, 5–6). As the obvious stages of cell cycle progression occurred in HeLa cells, most of the experiments related to cell cycle progression were performed not only in lung cancer cell lines but also in HeLa cells. Knockdown of USP24 increased the CCNA2 and E2F1 mRNA levels (Figure 4A); the same results were obtained in the cDNA array performed under USP24 knockdown conditions (Supplementary Figure 3A). In addition, knockdown of USP24 increased the G0/G1 transition into S-phase (Figure 4B; Supplementary Figure 3B). We analyzed the promoter-binding motif(s) of E2F1 and CCNA2, and found that the E2F-binding motif was present in the promoters of both E2F1 and CCNA2 (Supplementary Figure 3C). Overexpression of E2F4 in USP24-silenced cells rescued only the E2F1 mRNA level and partially abolished the effect of the USP24 knockdown on the G1–S transition and colony formation (Figures 4C–E; Supplementary Figure 3D), implying that E2F4 might be involved in USP24-mediated cell cycle progression. Knockdown of USP24 decreased the E2F4, p130 and TFDP1 levels, which form a complex to regulate the G1–S transition, but did not alter the E2F4 and TDFP1 mRNA levels (Figure 4F; Supplementary Figures 3E–G). We also examined the Rb level, which was reported to form a complex with E2F4; however, the Rb level was not affected by USP24 knockdown (Supplementary Figure 3H). Further study indicated that USP24 could interact with E2F4 (Figure 4G). Knockdown of USP24 decreased E2F4 protein stability (Figure 4H; Supplementary Figure 3I). The *in vitro* enzymatic assay using USP24 showed a decrease in the E2F4 ubiquitination signal in the presence of USP24 (Figure 4I), which provided direct evidence that E2F4 was a substrate of USP24. Conversely, knockdown of USP24 in A549 cells increased the E2F4 ubiquitination signal (Figure 4I). Taken together, our results demonstrated that USP24 knockdown decreased E2F4, p130 and TFDP1, resulting in an increase in the E2F1 levels and a subsequent increase in the G1–S transition, leading to tumorigenesis.

### The decrease in USP24 during mitosis is crucial for the metaphase–anaphase transition via the declining securin level

In addition to studying the effect of USP24 on the G1–S transition, we studied the role of USP24 on other stage(s) of cell cycle progression (Figures 5 and 6). Knockdown of USP24 increased the CDK1 and cyclin B1 levels, and increased the ratio of cells in the G2/M stage, indicating that USP24 reduction was beneficial for cell cycle progression (Supplementary Figures 4A and B). USP24 was significantly decreased in mitotic cells (Figures 5A–C; Supplementary Figures 4C and D). We found that the USP24 protein stability was higher in interphase than in mitosis (Figure 5D). MG132 treatment rescued the USP24 level in the mitotic stage (Supplementary Figure 4E). Moreover, the USP24 ubiquitination signal was increased during the mitotic stage (Figure 5E; Supplementary Figure 4F), implying that the decrease in the USP24 level during mitosis might be due to the enhancement of USP24 degradation. In addition, there was an inverse correlation between the USP24 level and the cdc20 level (an E3 ligase in the APC/C complex), but not cdh1 level, implying that APC/C^cdc20^ might provide the enzymatic activity for USP24 ubiquitination (Figure 5F; Supplementary Figure 4G). Further study found that USP24 interacted with cdc20 during the mitotic stage (Figures 5g and h; Supplementary Figures 4H and I). Overexpression of GFP-USP24 increased the cyclin B1 level, suggesting that USP24 was beneficial for cells that remained in mitosis (Supplementary Figure 4J). To elucidate the detailed molecular mechanism underlying the effect of USP24 on mitotic progression, we examined the mitotic sub-phases. The results indicated that a higher percentage of cells stayed in metaphase under GFP-USP24 expression (Figure 6A). Previous studies have indicated that securin is crucial for the metaphase–anaphase transition. Here we found that USP24 interacted with securin in mitotic cells (Figure 6B; Supplementary Figure 4K). Nocodazole-treated cells that stayed in G2 phase exhibited increased securin level that quickly decreased after nocodazole release, implying that a decrease in the securin level was important for mitotic progression and the metaphase–anaphase transition (Supplementary Figure 4L). Knockdown of USP24 decreased securin expression and increased the securin ubiquitination signal, whereas USP24 overexpression increased securin expression (Figures 6C and D; Supplementary Figure 4M). The *in vitro* enzymatic assay using USP24 showed that the securin ubiquitination signal was decreased in the presence of USP24, suggesting that securin was a substrate of USP24 (Figure 6E). Taken together, our results suggested that USP24 was decreased by APC/C^cdc20^ to decrease the securin level, which was beneficial for the metaphase–anaphase transition and enhanced cell cycle progression.

### Phosphorylation of USP24 increases its degradation during tumorigenesis and cell cycle progression

**Figure 7:**
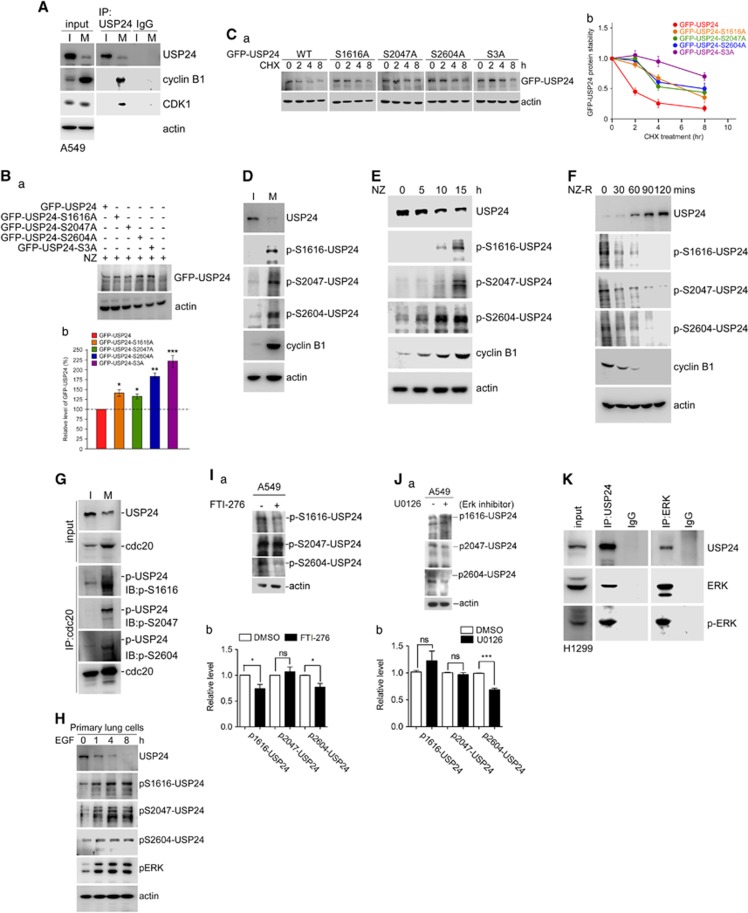
Regulation of USP24 by phosphorylation increases its degradation. (**A**) Samples were collected from A549 cells that remained in interphase (I) and mitosis (M) for the immunoprecipitation assay with an anti-USP24 antibody. The immunoprecipitated (IP) samples were analyzed by western blotting with antibodies against USP24, CDK1, cyclin B1 and actin. (**B**) Various mutations of USP24 phosphorylation residues, including a triple mutant (S3A), were constructed and expressed in cells. Samples were analyzed by western blotting with an anti-GFP antibody (a). The GFP-USP24 level was quantified after three independent experiments (b). (**C**) The proteins stabilities of USP24-wt (WT) and its mutants (S1616A, S2047A, S2604A and S3A) were investigated after cycloheximide (CHX) treatment. Samples were analyzed by western blotting with an anti-GFP antibody (a). Proteins levels were quantified after three independent experiments (b). (**D**) Normal or mitotic A549 cells were collected for an immunoblotting assay with antibodies against USP24, phospho-USP24 (S1616), phospho-USP24 (S2047), phospho-USP24 (S2604), cyclin B1 and actin. (**E**, **F**) Samples were collected under nocodazole treatment (NZ) (**E**) or nocodazole release (NZ-R) (**F**). Samples were analyzed with antibodies against USP24 phosphorylation at indicated residues, cyclin B1 and actin. (**G**) A549 cells treated with nocodazole were collected for an immunoprecipitation assay with anti-cdc20. The immunoprecipitated samples were used for western blotting with antibodies against the indicated proteins. (**H**) Samples collected from EGF-treated mice lung primary cells were analyzed by western blotting with antibodies against indicated proteins. (**I**) Samples were collected from A549 cells treated with FTI-276, and then analyzed by western blotting with antibodies against USP24 phosphorylation at the indicated residues (a). The relative levels were quantified after three independent experiments (b). (**J**) Samples were collected from A549 cells treated with Erk inhibitor, and then analyzed by western blotting with antibodies against USP24 phosphorylation at the indicated residues (a). The relative levels were quantified after three independent experiments (b). (**K**) Samples were collected from H1299 cells for immunoprecipitation with anti-USP24 and anti-ERK antibodies, and then immunoprecipitated samples were used to study the USP24, Erk and p-Erk levels by western blotting with anti-USP24, anti-Erk and anti-p-Erk antibodies.

**Figure 8:**
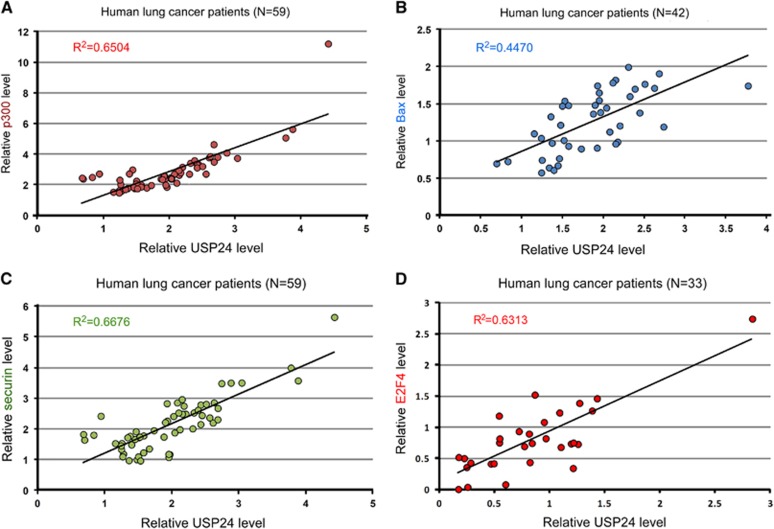

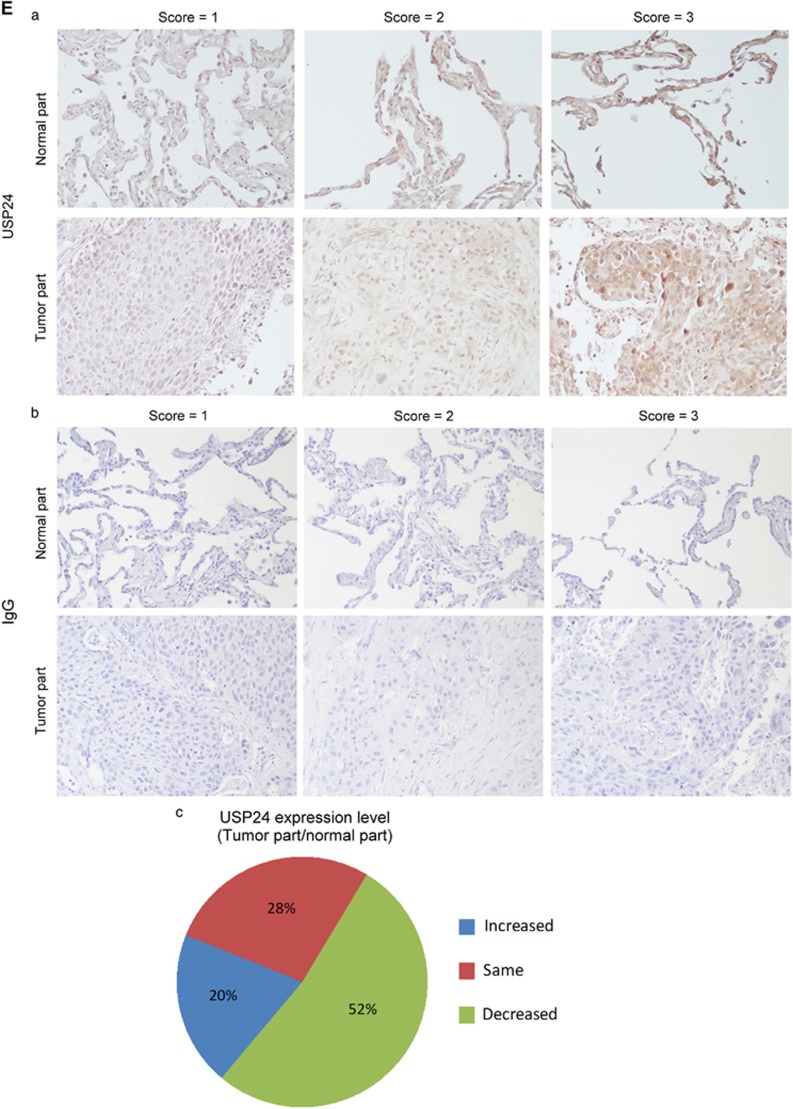
USP24 expression correlated with its substrates in clinical lung cancer samples. (**A**–**D**) USP24 expression correlated with its substrates, p300 (**A**), Bax (**B**), Securin (**C**) and E2F4 (**D**), in clinical lung cancer samples. (**E**) The USP24 level was examined in the specimens from normal lung tissue and lung cancer patients using immunohistochemistry with an anti-USP24 antibody (a) or IgG as an internal control (b). USP24 expression between lung cancer patients and the corresponding normal lung tissue (c).

Next, we examined the mechanism by which USP24 expression was regulated during cell cycle progression and tumorigenesis (Figure 7). We found that USP24 interacted with CDK1/cyclin B during the mitotic stage, and that the USP24 phosphorylation signal was higher in mitosis than in interphase (Figure 7A; Supplementary Figure 5A). Our previous liquid chromatography–tandem mass spectrometry (LC/MS/MS) studies probed several phosphorylation residues within USP24 (Supplementary Table 1). The phosphorylation residues were mutated individually; then, we investigated the expression of USP24-wt and its mutants (Figures 7B and C; Supplementary Figure 5B). Three mutated residues and triple mutant S3A increased the USP24 level, suggesting that USP24 phosphorylation might be the reason for the decreased USP24 expression during mitosis and tumorigenesis. Mutations at these residues increased the USP24 half-life during mitosis, suggesting that USP24 phosphorylation during mitosis decreased USP24 expression (Figure 7D). Antibodies that recognized these phosphorylation residues were produced to study USP24 phosphorylation. The specificities of the phosphorylation-specific antibodies were assessed by measuring their recognition of the phospho-peptide but not the peptide alone (Supplementary Figure 5C). USP24 knockdown also decreased the antibody signals (Supplementary Figures 5D and E). Phosphorylation of USP24 at these three residues was increased during mitosis (Figures 7E and F). Moreover, cdc20 could also interact with USP24 when these residues were phosphorylated, implying that the decrease in USP24 during mitosis might be important for cell cycle progression and cancer cell proliferation (Figure 7G). Furthermore, the data in Figure 1 indicated that USP24 expression was also negatively regulated by EGFR- and Kras-activated signaling pathways. Here we also found that USP24 phosphorylation at Ser1616, Ser2047 and Ser2604 was increased in EGF-treated primary lung cells (Figure 7H). Conversely, USP24 phosphorylation at Ser1616 and Ser2604 was decreased in FTI-276-treated A549 cells (Figure 7I). In addition, we found that USP24 phosphorylation at Ser2604 was declined by U0126, which inhibited the Erk1/2 activities in A549 cells (Figure 7J); however, USP24 phosphorylation was not decreased by LY294002, which inhibited the PI3K activities (Supplementary Figure 5F). Further study indicated that USP24 could interact with Erk1/2 and p-Erk1/2 (Figure 7K), suggesting that Erk1/2 might be the kinase for phosphorylation of USP24 at S2604 to decrease the USP24 level. Finally, we used samples from clinical lung cancer patients to study the relevance between USP24 and its substrates (Figures 8A–D). The data indicated that there was a high relevance between the USP24 level and the substrates identified in this study, suggesting that USP24 might regulate p300, Bax, E2F4 and securin levels to regulate apoptosis and cell cycle progression, resulting in cancer formation. However, we did not find a good correlation between the mRNAs of USP24 and its substrates, indicating that USP24 was a deubiquitinase that stabilized its substrates (Supplementary Figures 6A–D). Furthermore, we investigated the clinical relevance of USP24 between normal lung tissue and lung cancer specimens for immunohistochemistry staining with an antibody against USP24 (Figure 8E). These results indicated that USP24 expression was downregulated in lung adenocarcinoma.

## Discussion

**Figure 9:**
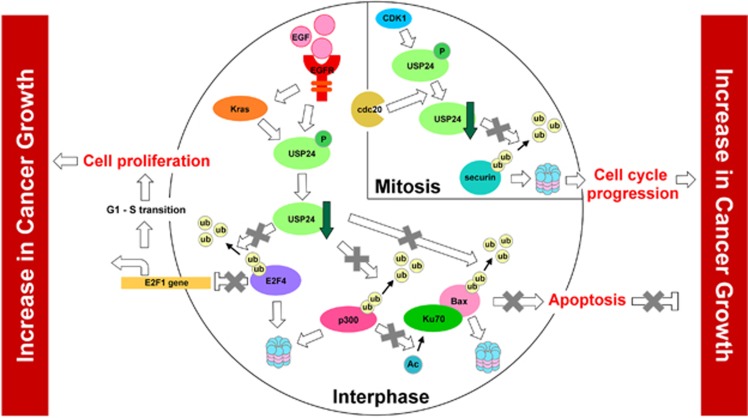
Scheme illustrating the mechanistic model of EGF-mediated USP24 downregulation in increase cancer growth.

In this study, we found that the phosphorylation of USP24 by CDK1- or EGF-mediated kinase activity decreased the USP24 level. In turn, this decrease in USP24 expression reduced the levels of several proteins related to cancer formation and were shown to be substrates of USP24. The reductions in the expression levels of these proteins decreased apoptosis and increased cell cycle progression, leading to positive regulation of cancer formation. Our data support that, together with other oncogenes such as Kras or EGFR, USP24 downregulation positively affected the efficacy of lung cancer formation (Figure 9).

Here we found an inverse correlation between the USP24 level and EGFR mutations in lung cancer clinical samples, suggesting that USP24 might negatively regulate cancer formation. Here we found that the phosphorylation of several USP24 residues was regulated by EGF treatment and mitotic CDK1 to enhance USP24 degradation in a polyubiquitination-dependent manner. USP24 phosphorylation increased the recruitment of the E3 ligase cdc20, which is the subunit of the APC/C complex that provides the E3 ligase activity. Because transcriptional activity is attenuated during mitosis, the regulation of protein turnover is a very crucial issue for the maintenance of mitotic progression. Many studies have indicated that a combination of post-translational modification and protein ubiquitination is the major strategy to control protein levels during mitosis.^22^ Here we added one new member (USP24) that deubiqutinated securin to regulate cell cycle progression.

Our previous study indicated that USP24 variants had increased protein levels due to increased RNA stability.^12^ In this study, we found that the modification of USP24 by phosphorylation decreased its protein stability in Kras^G12D^- and EDFR^L858R^-induced lung cancer mouse models, lung cancer cell lines and mitotic cells. Based on the data in this study and our previous study, we propose that EGFR^L858R^- and Kras^G12D^-mediated signaling pathways inhibit USP24 expression, which is beneficial for cancer formation during the early stage. Although early-stage lung cancer patients with EGFR mutations exhibit a higher correlation with lower USP24 expression, other patients (especially at the late stage) express higher levels of USP24. Our recent study indicated that USP24 overexpression during the late stage contributed to lung cancer malignancy.^12^ In addition, we found that USP24 variants contributed to USP24 upregulation, thereby leading to cancer malignancy. In addition to the effect of the variants on USP24 expression, our preliminary results showed that transforming growth factor-β treatment increased USP24 expression. Based on these findings, we propose that USP24 negatively regulates cancer tumorigenesis but positively controls cancer malignancy. Furthermore, we uncovered the mechanisms controlling USP24 regulation during cancer progression.

Two effects (apoptosis inhibition and increased proliferation) contribute to tumorigenesis. Therefore, we studied the effect of USP24 on apoptosis and proliferation. Previous studies show that Ku70 acetylation facilitates the mitochondria localization of Bax, thus induces apoptosis.^14^ In this study, we found that USP24 downregulation decreased the p300 level, thereby decreasing Ku70 acetylation. These effects resulted in the inhibition of Bax transportation to the mitochondria, finally leading to the inhibition of cell apoptosis. In addition to the role of Ku70 in apoptosis, most previous studies related to Ku70 demonstrated its involvement in DNA damage repair activities.^23, 24^ Therefore, USP24 might also be involved in DNA damage repair activity. In addition, USP24 also directed Bax protein stability to result in cell apoptosis. When investigating the role of USP24 in apoptosis, we identified two crucial proteins (p300 and Bax) as USP24 substrates. Therefore, during the initiation of tumor formation, EGFR mutations decrease USP24 to increase the degradation of p300 and Bax, and thus repress cell apoptosis. Previous studies indicated that p300 was a tumor suppressor and Bax was an apoptotic inducer that prevented cancer formation.^25, 26^ Although their levels were increased in some specimens from late-stage lung cancer patients, inhibition of Kras^G12D^- or EGFR^L858R^-mediated activities in all cell lines at different stages increased the USP24 level. Other factor(s) in addition to the EGFR-activated pathway may be involved in the regulation of USP24 during tumorigenesis. For example, we found that transforming growth factor-β treatment also increased the USP24 level.

Phosphorylation of USP24 increases its degradation during tumorigenesis and cell cycle progression. Because protein translation is nearly attenuated during mitosis, post-translational modification of proteins including phosphorylation is a critical strategy for maintaining protein levels. Our recent study found >10 000 phosphorylation residues localized within thousands proteins by LC/MS/MS; these phosphorylation events might regulate the activities and levels of proteins to regulate mitosis progression. Several phosphorylation residues in USP24 are involved in the control of protein stability by increasing the interaction between USP24 and the APC/C complex, suggesting that APC/C might be important for the downregulation of USP24. Therefore, APC/C downregulates the securin level through not only its E3 ligase function on securin but also its E3 ligase on USP24, which allows securin to be more efficiently downregulated and results in the complete and rapid metaphase–anaphase transition. In addition to the phosphorylation of USP24 in mitosis, USP24 is also phosphorylated by EGF-activated Erk1/2 kinase activity to turnover USP24. Furthermore, Ser2047 and Ser2604 are as typical consensus sites (serine–proline) for CDK and ERK; however, Ser1616 might be as atypical. Finally, many diseases are caused by cell cycle progression disorders. For example, the EGFR mutation and CDK1-mediated kinase activities contribute to USP24 downregulation, resulting in cancer formation by affecting its substrates. This study provides important findings for USP24-mediated protein turnover including p300, Bax, E2F4 and securing to regulate apoptosis and proliferation of cancer cells, respectively, which is crucial for cancer tumorigenesis. Understanding protein turnover will be beneficial for disease prevention in the future.

## Materials and methods

### Cell culture and transfection

Human lung adenocarcinoma epithelial cell line A549 was cultured in RPMI 1640 medium (Invitrogen, Carlsbad, CA, USA), and human non-small cell lung carcinoma cell line H1299, human cervical adenocarcinoma cell line HeLa and human epithelial carcinoma cell line A431 were cultured in low-glucose Dulbecco's modified Eagle's medium (DMEM; Thermo Scientific, Waltham, MA, USA), and human osteosarcoma cell line U2OS was cultured in McCoy's 5A medium (Invitrogen). McCoy's 5A, low-glucose DMEM and RPMI medium contain 10% fetal bovine serum, 100 μg/ml streptomycin sulfate and 100 U/ml penicillin G sodium. All cell lines were maintained at 37 °C and 5% CO_2_. A549 and H1299 cells were identified and authenticated by Food and Industry Research and Development Institute (Hsinchu, Taiwan). Normal mouse primary lung cells were isolated from lungs homogenized by using 0.5% collagenase (Sigma-Aldrich, St Louis, MO, USA) and 0.05% trypsin (Biological Industries, Kibbutz Beit Haemek, Israel) at 37 °C for 1 h. After centrifuging at 1200 *g* for 20 min, and filtering, cells were resuspended by DMEM-10% FBS and seeded onto six-well plates. Cells (2.5 × 10^5^) were seeded on a six-well plate and were then transfected when they reached 40–50% confluence with plasmids using Lipofectamine 2000 (Invitrogen) or PolyJet (SignaGen Laboratories, Rockville, MD, USA) in accordance with the manufacturer's instructions.

### Colony formation assay

A549 cells were infected with lentivirus-expressing scramble and shUSP24 shRNA for 3 days and seeded in six-well plates at a density of 5 × 10^3^ per well. After 2 weeks of culture, cells were washed with phosphate-buffered saline and colonies were visualized by incubating with 2% methylene blue (Sigma-Aldrich) for 30 min. After 30 min of incubation, methylene blue was removed and colonies were washed with distilled water for 3 times. ImageJ (NIH, Bethesda, MD, USA) were used to perform the statistical analysis of colony numbers.

### MTT cell viability assay

A549 cell infected with lentivirus-expressing scramble or shUSP24 for 3 days and seeded in 96-well plates (1 × 10^4^ cells per well) for 24 h. Cell viability was examined using the 3-(4,5-Dimethylthiazol-2-yl)-2,5-Diphenyltetrazolium Bromide (MTT) assay kit according to the manufacturer's instruction (Biovision Inc., Milpitas, CA, USA).

### *In vitro* deubiquitination assay

Cell lysates with MG132 (5 μm) treatment were immunoprecipitated with Bax, p300, securin or E2F4 antibody for 4 h, and then incubated with protein A-Sepharose for 1 h. After washing, recombinant human USP24 (Origene, Rockville, MD, USA) was added to the substrates for 2 h at 37 °C in deubiquitination buffer (50 mm Tris (pH 8.0), 10 mm dithiothreitol and 5 μm MG132). Reactions were stopped by adding 2 × sample buffer and analysed using western blotting.

### Fluorescence-activated cell sorting analysis

The cells washed by cold phosphate-buffered saline, and then fixed in 70% alcohol at 4 °C for 16 h. After fixation, the cells washed by cold phosphate-buffered saline and treated with 0.1% TritonX-100 for 10 min for permeation. Permeable cells were treated with 10 μg/ml RNase (Qiagen, Frederick, MD, USA) and 50 μg/ml propidium iodide for 1 h. The cells were analysed by flow cytometer (Cell Lab Quanta SC; Beckman Coulter, Brea, CA, USA).

### Study approval

Human study has been approved by the Institutional Review Board (IRB) on 9 January 2014 and the valid execution date is from 1 August 2014 to 31 July 2017 (IRB no: B-ER-102-400). The IRB of National Cheng Kung University Hospital is organized and operated according to the laws and regulations of International Council for Harmonization-Good Clinical Practice. Animal study has been approved by the Institutional Animal Care and Use Committee, and the valid execution date is from 1 August 2014 to 31 July 2017 (Institutional Animal Care and Use Committee approval no: 103117).

### Collection of specimens from lung cancer patients

The study using human specimens was approved by the Clinical Research Ethics Committee at National Cheng Kung University Medical Center (Tainan, Taiwan). After surgical resection at National Cheng Kung University Hospital, specimens of patients with lung adenocarcinomas were collected for Immunohistochemical analysis or western blotting. The pathological data were analyzed by clinical pathologists.

### Statistics

All samples or animals (not randomized) were used for statistical analysis. The difference between two groups was analyzed by Student's *t*-test. Kaplan–Meier method was used to evaluate the survival curve, and the comparison of two survival curves was analyzed by log-rank test. The *P*-value, which is <0.05, was considered as statistically significant. S.e.m. is used to calculate and plot error bars from raw data.
