# Supplementary figures and images for: EGF-mediated inhibition of ubiquitin-specific peptidase 24 expression has a crucial role in tumorigenesis
Source: Oncogene. 2016 Dec 19;36(21):2930–45. doi: 10.1038/onc.2016.445 (PMC5454318; doi:10.1038/onc.2016.445)

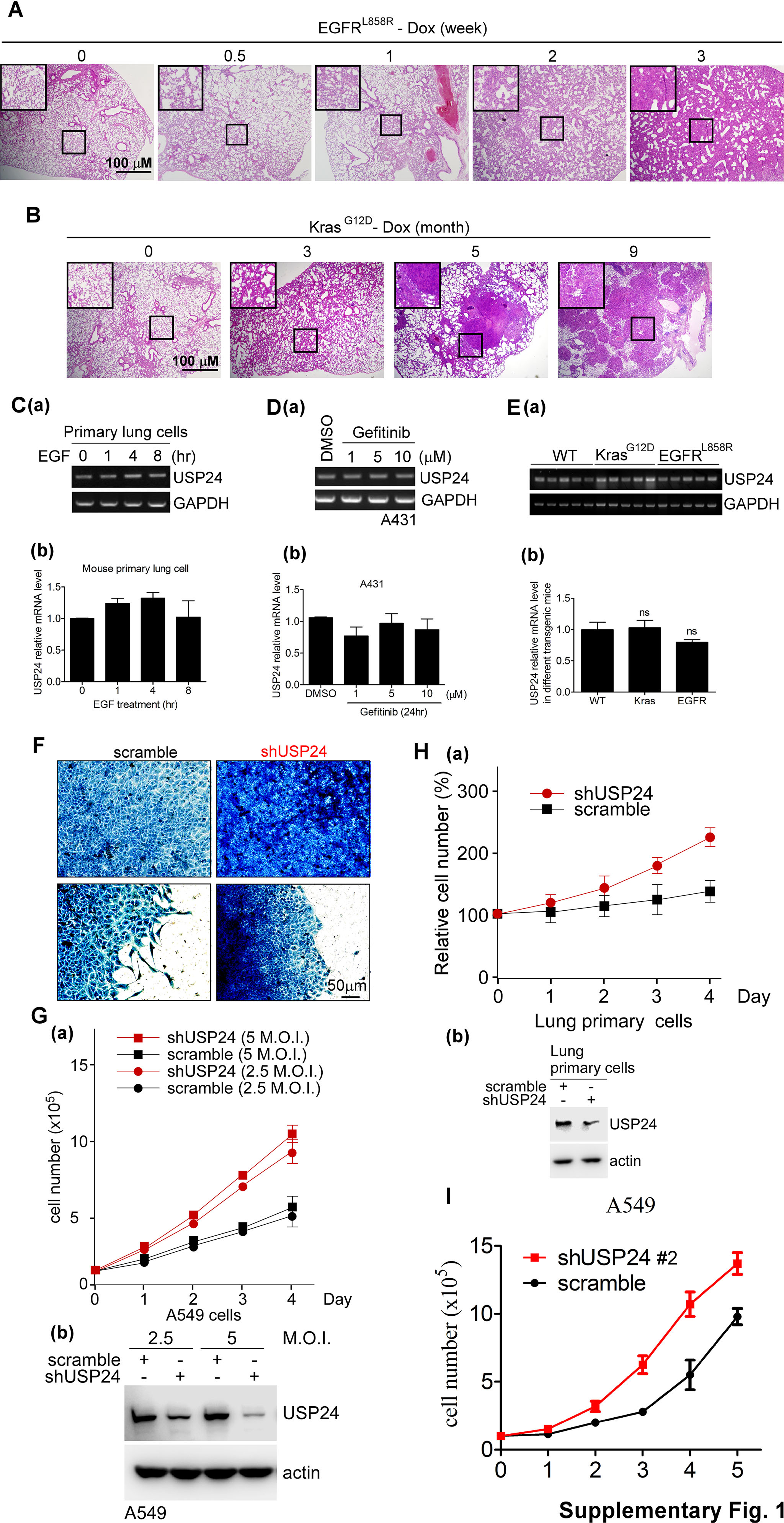

Supplement: Supplementary Figure 1 [file onc2016445x1.tif]

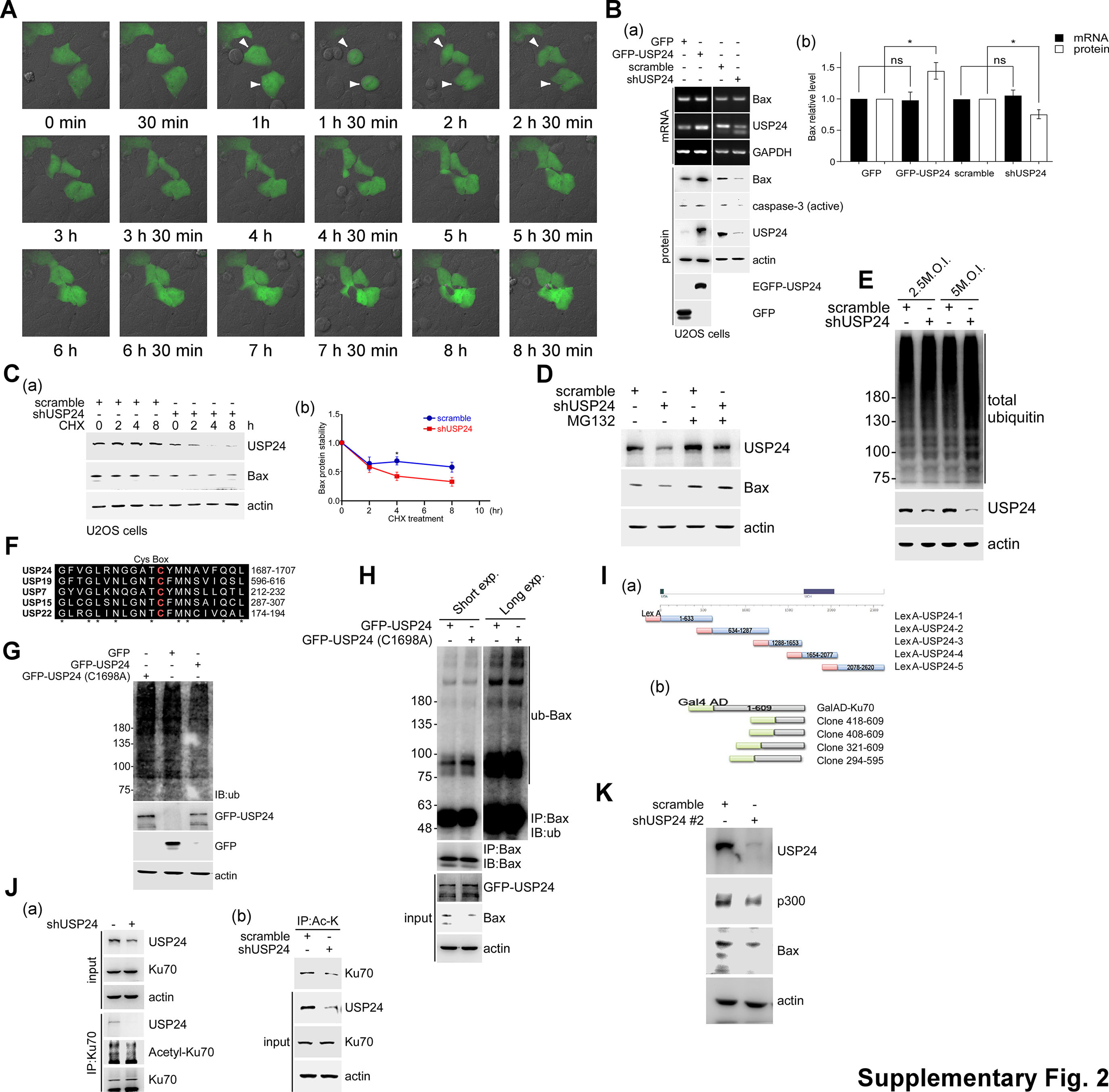

Supplement: Supplementary Figure 2 [file onc2016445x2.tif]

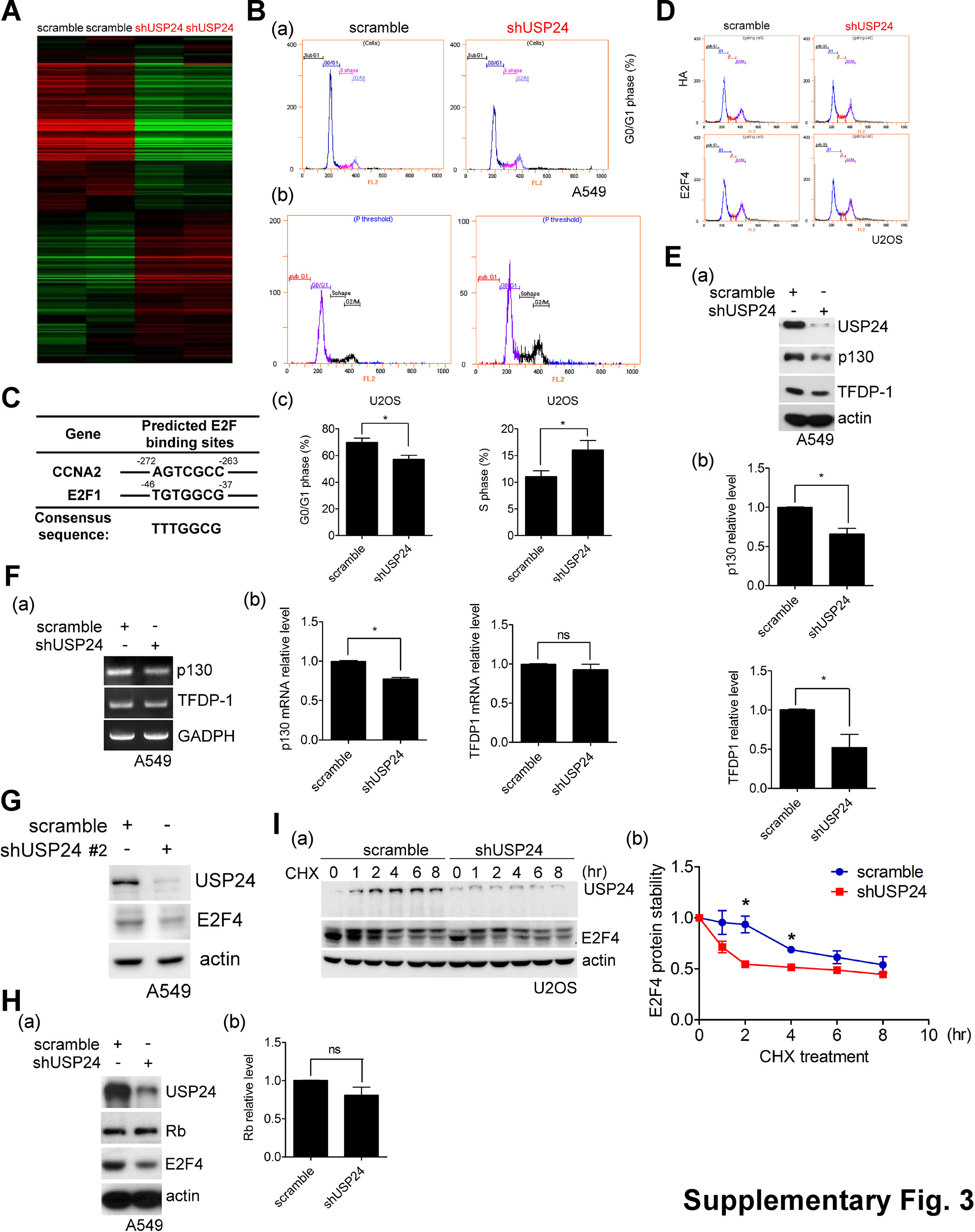

Supplement: Supplementary Figure 3 [file onc2016445x3.tif]

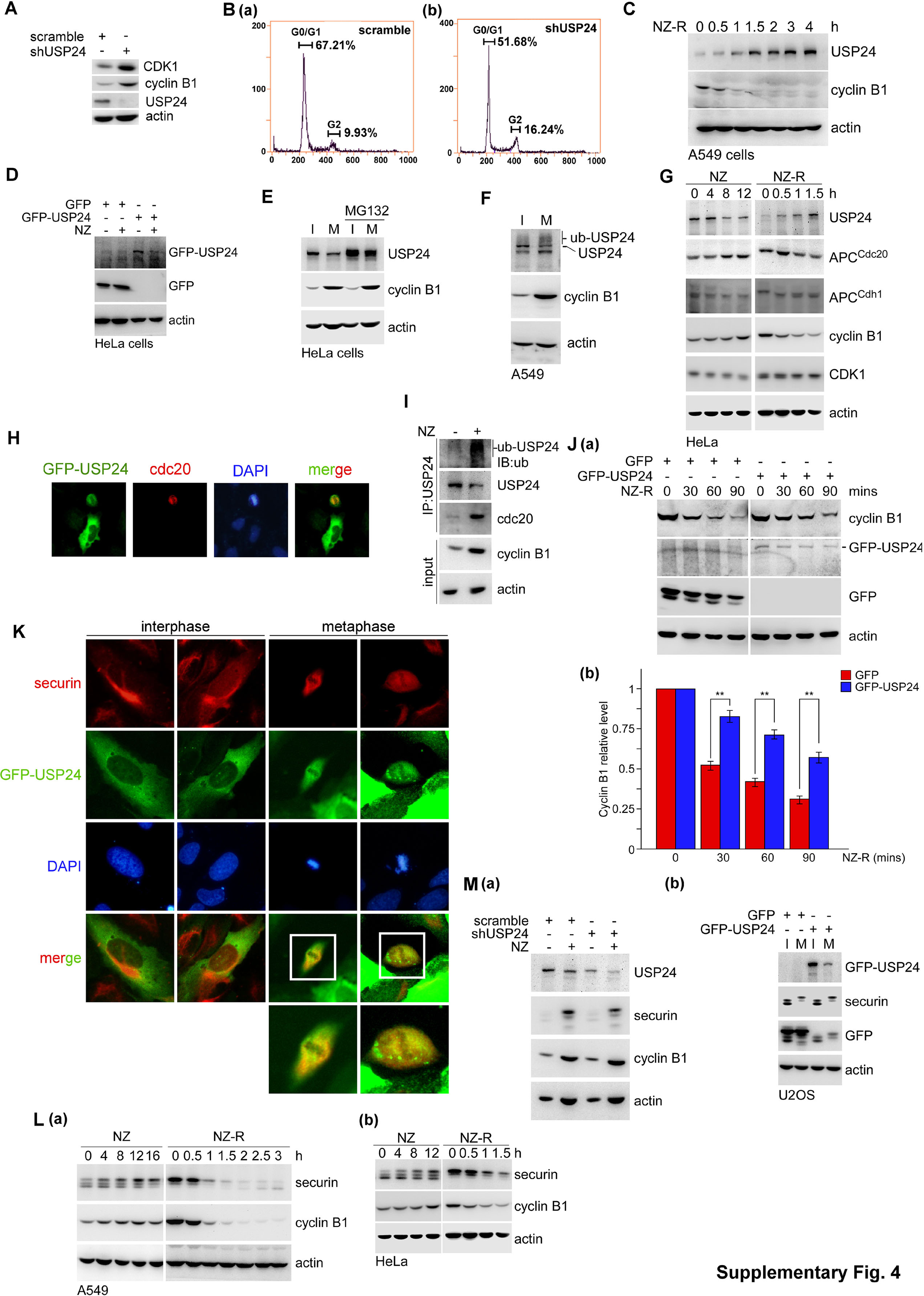

Supplement: Supplementary Figure 4 [file onc2016445x4.tif]

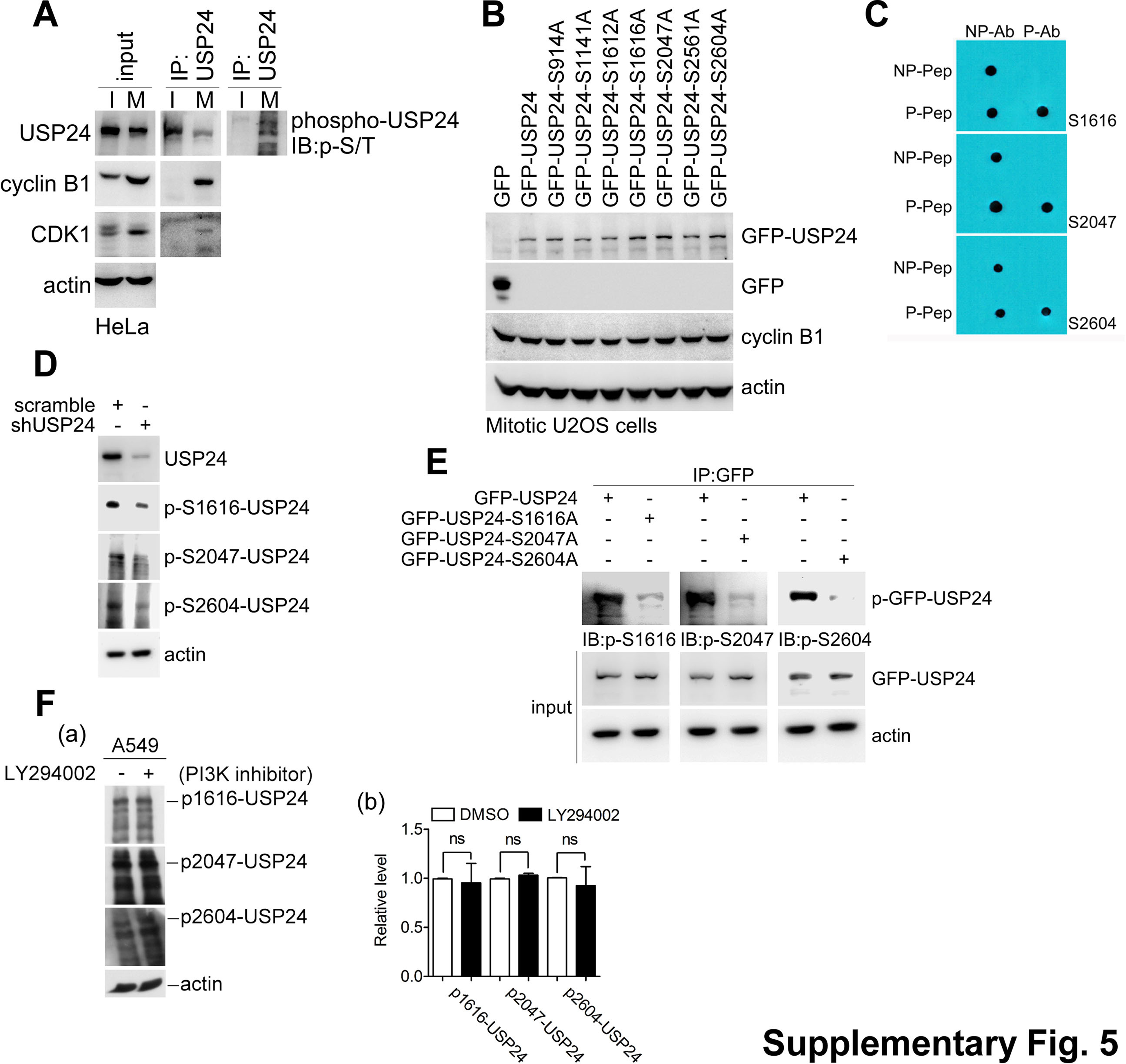

Supplement: Supplementary Figure 5 [file onc2016445x5.tif]

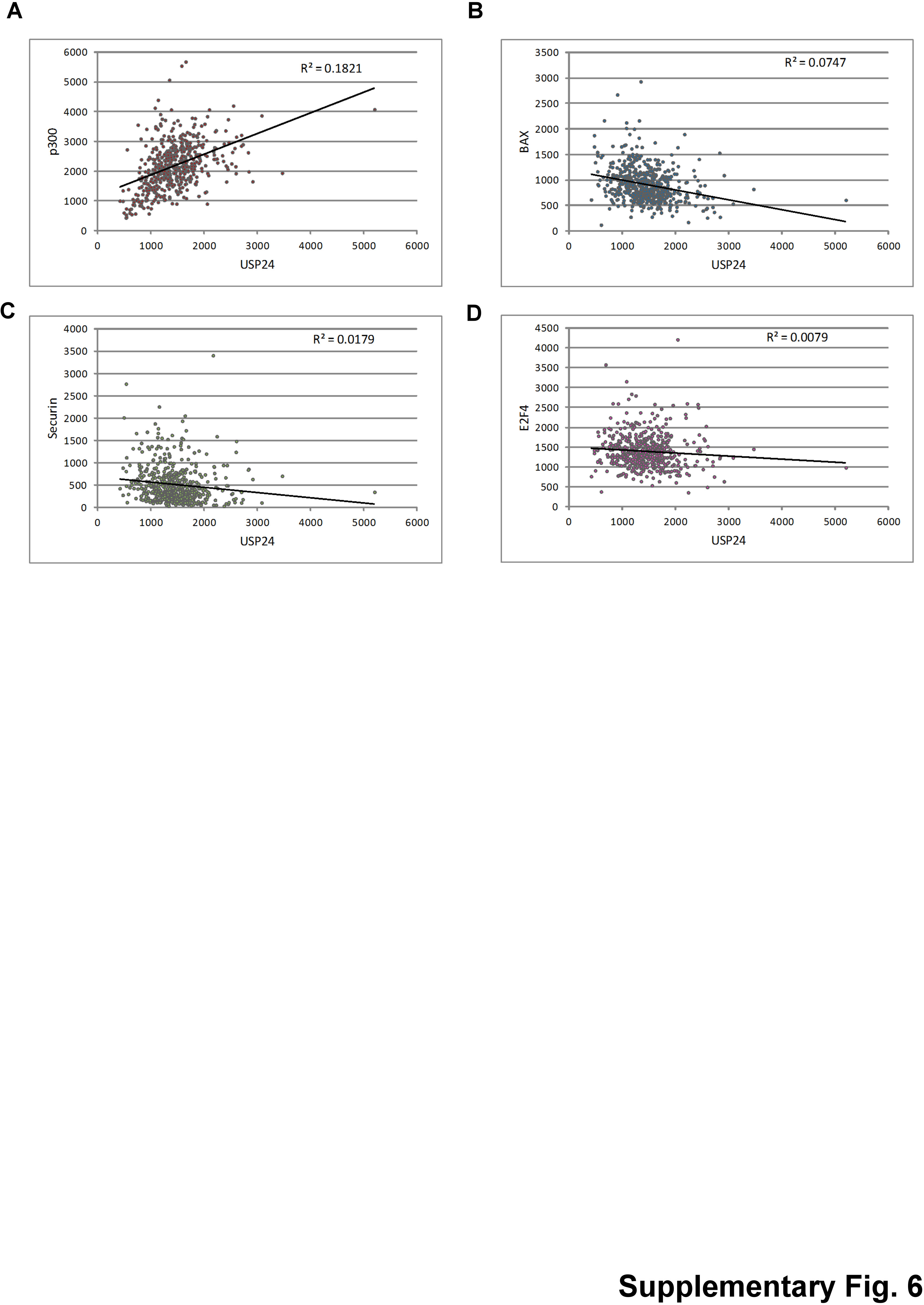

Supplement: Supplementary Figure 6 [file onc2016445x6.tif]
