# Supplementary material for: EGF-mediated inhibition of ubiquitin-specific peptidase 24 expression has a crucial role in tumorigenesis
Source: Oncogene. 2016 Dec 19;36(21):2930–45. doi: 10.1038/onc.2016.445 (PMC5454318; doi:10.1038/onc.2016.445)
Supplement: Supplementary Tables [file onc2016445x7.docx]

**EGF-mediated Inhibition of Ubiquitin-specific Peptidase 24 Expression Plays a Crucial Role in Tumorigenesis**

Shao-An Wang, Yi-Chang Wang, Yun-Pei Chuang, Yi-Han Huang, Wu-Chou Su, Wen-Chang Chang, Jan-Jong Hung

**Supplementary tables**

**Supplementary Table 1.** Several residues within USP24 were determined by LC/MS/MS.


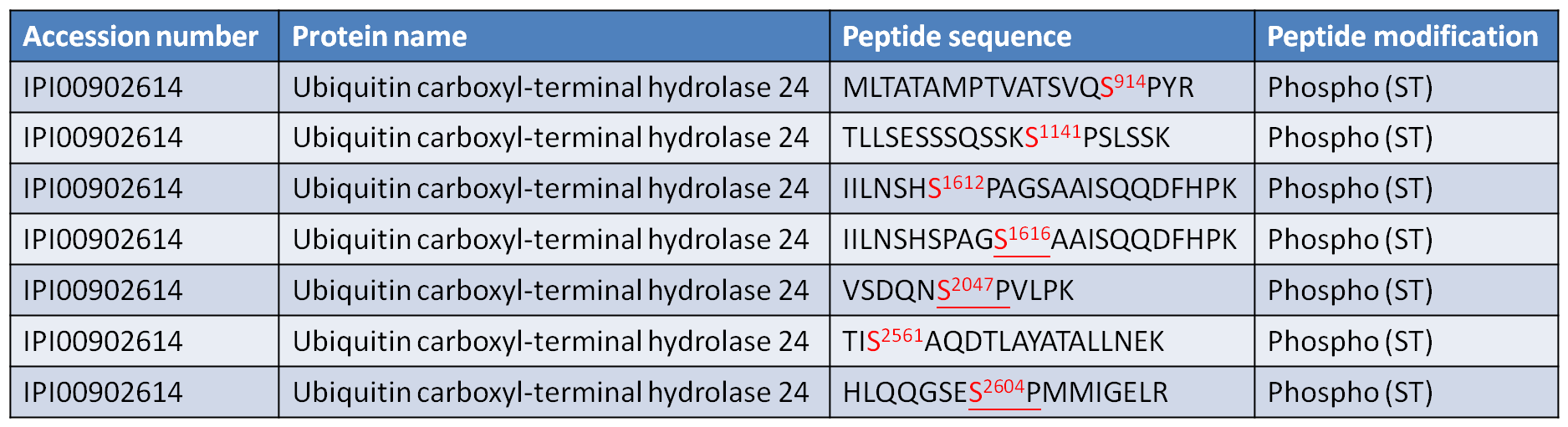


**Supplementary Table2.** Antibodies used in western blot.

| **Protein** | **Brand** | **Protein** | **Brand** |
| --- | --- | --- | --- |
| actin | Sigma-Aldrich | p300 | BD |
| USP24 | Proteintech | Acetyl-lysine | Cell Signaling |
| EGFR L858R | Cell Signaling | E2F4 | Abcam |
| Erk | Millipore | Rb | Genetex |
| pERK | Cell Signaling | p130 | Genetex |
| pEGFR | Cell Signaling | TFDP-1 | Genetex |
| Bax | Proteintech | Cyclin B1 | Santa Cruz |
| Caspase-3 | Cell Signaling | CDK1 | Santa Cruz |
| Ubiquitin | Genetex/Santa Cruz | Cdc20 | Proteintech |
| GFP | Santa Cruz | Securin | Abcam |
| Ku70 | Abcam | Phospho-serine/threonine | Cell Signaling |
| HA | Roche | p-S1616/S2047/S2604-USP24 | Kelowna |
| COX IV | Novus Biologicals |  |  |

**Supplementary Table 3.** Primers used in PCR, and Q-PCR

| Gene | Forward (5’ 🡪 3’) | Reverse (5’ 🡪 3’) |
| --- | --- | --- |
| p300 | AAATACTGCTCCAAGCTC | TCCAATTAGGATTTCATG |
| Bax | TTTGCTTCAGGGTTTCATCC | CAGTTGAAGTTGCCGTCAGA |
| CCNA | TTATTGCTGGAGCTGCCTTT | CTCTGGTGGGTTGAGGAGAG |
| GAPDH | GAGTCAACGGATTTGGTCGT | TTGATTTTGGAGGGATCTCG |
| E2F1 | ATGTTTTCCTGTGCCCTGAG | ATCTGTGGTGAGGGATGAGG |
| USP24 | CAGTTGTGCTCTCCTGTGGA | AGGGATTTCTCCTGCTCCAT |
| p130 | ATTTGGCATGGAAACCAGAG | GTCACCCTTCTGGGAGTCAA |
| TFDP1 | TCGTCAACACCAGCAAGAAG | TGGCCATTTTAAGGTCTTCG |

**Supplementary Table 4.** Primers for GFP-USP24 expression plasmid construction.

| USP24-1 | Forward: 5’-AGATCTAATGGAATCGGAGGAGGA-3’ |
| --- | --- |
|  | Reverse: 5’-GGTACCACCCATACAAACTGGGGA-3’ |
| USP24-2 | Forward: 5’-GGTACCAGCTTTGCGTCAGCTCCA-3’ |
|  | Reverse: 5’-GGTACCACATAAGGACATCTGTCT-3’ |
| USP24-3 | Forward: 5’-GGTACCCCAGAAAAGTCATCCTAC-3’ |
|  | Reverse: 5’-GGATCCTCCACCGCCCTGATCCAC-3’ |
| USP24-4 | Forward: 5’-GGATCCCCACGAAAAAAGGTTGCC-3’ |
|  | Reverse: 5’-GGATCCCTAGGGATCAACATCATC-3’ |
