# Supplementary material for: EGF-mediated inhibition of ubiquitin-specific peptidase 24 expression has a crucial role in tumorigenesis
Source: Oncogene. 2016 Dec 19;36(21):2930–45. doi: 10.1038/onc.2016.445 (PMC5454318; doi:10.1038/onc.2016.445)
Supplement: Supplementary Materials [file onc2016445x8.docx]

**EGF-mediated Inhibition of Ubiquitin-specific Peptidase 24 Expression Plays a Crucial Role in Tumorigenesis**

Shao-An Wang, Yi-Chang Wang, Yun-Pei Chuang, Yi-Han Huang, Wu-Chou Su, Wen-Chang Chang, Jan-Jong Hung

**Supplementary Materials and Methods**

**Western Blotting**

Cell lysates were prepared from the indicated cell lines for SDS-polyacrylamide gel electrophoresis (SDS-PAGE), which was then transferred to a polyvinylidene difluoride membrane (Millipore, Billerica, MA, USA) by using a transfer apparatus according to the manufacturer’s protocols. Membranes were blocked with 3% nonfat milk in TBST buffer (10 mM Tris-HCl, pH 8.0, 150 mM NaCl and 0.05% Tween 20) for 1 h, washed in the same buffer and incubated with antibodies at 4°C overnight. Membranes were washed three times for 10 min and incubated with the secondary antibody (goat anti-rabbit or anti-mouse immunoglobulin G linked with horse radish peroxidase (Millipore)) for 1 h at room temperature. After three more washes, the protein bands were detected with the ECL Western blotting Detection System (Millipore) and recorded with the FluorChem image analysis system (Alpha Innotech, San Leandro, CA, USA). Band intensities were quantified with Scion image software (Scion, Frederick, MD, USA). Brands of antibodies were described as Supplementary Table 2.

**Reverse Transcription-Polymerase Chain Reaction (RT-PCR)**

Total RNA of cells was isolated with a TRIsure RNA extraction kit (Bioline, Taunton, MA, USA), and 3 μg of RNA was subjected to RT-PCR with SuperScript III (Invitrogen). PCR products were separated by 1% agarose gel electrophoresis and visualized by ethidium bromide staining. Primers sequences were described as Supplementary Table 3.

**Transgenic Mice**

Transgenic mice were acquired from Jackson Lab (Bar Harbor, MA, USA) and maintained at the National Laboratory Animal Center in Taiwan. Reverse tetracycline trans-activator (rtTA) protein was expressed under the control of Scgb1a1 (secretoglobin, family 1A, member 1) promoter in Scgb1a1-rtTA transgenic mice. To generate the activated Kras (Kras4b^G12D^) in TetO-Kras4b^G12D^ transgenic mice, tetracycline-responsive promoter element (TRE; tetO) was utilized. The TetO-EGFR^L858R^ transgenic mice expressed EGFR^L858R^ was also under the regulation of a tetracycline-responsive promoter element (TRE; tetO). Both Kras4b^G12D^ and EGFR^L858R^ were crossed with Scgb1a1-rtTA transgenic mice to generate bitransgenic mice. In order to induce the generation of Kras4b^G12D^ and EGFR^L858R^ in bitransgenic mice, doxycycline (0.5g/l) was added to the drinking water, starting at the age of 6 weeks.

**Cell Synchronization**

Mitotic cells were collected by incubating A549 or HeLa cells in complete medium with 45 ng/ml nocodazole at 37 °C, and the cells were collected after different time intervals and then prepared for immunoblotting. For another kind of cell synchronization, mitotic cells were collected by incubating A549 or HeLa cells in complete medium with 45 ng/ml nocodazole at 37 °C for 16 h. Cells were then washed three times with PBS and added with fresh medium. The releasing cells were then collected after different time intervals and lysed in RIPA buffer as described above. Equal amounts of proteins from these cell extracts were analyzed using immunoblotting.

**Immunohistochemistry**

Human specimens were incubated in 10 % formaldehyde for 24 hours for fixation, dehydration, and embedded in paraffin. Hematoxylin and eosin were used for staining sections (5 mm). For immunohistochemistry, paraffin-embedded sections were incubated in xylene for dewaxing and a graded series of ethanols for dehydration. Sections were incubated in PBS with 0.3% hydrogen peroxide for 30 minutes to block endogenous peroxidase, and then incubated in PBS with 1 % bovine serum albumin for blocking. USP24 primary antibody (1:200) was used to cover sections for 1 hour in room temperature, and Vectastain ABC kit (Vector Laboratories, Burlingame, CA, USA) was used for visualize the immunoreactivity. Sections were photographed by Olympus BX-51 microscope (Olympus, Tokyo, Japan).

**Immunoprecipitation**

Cell extracts were prepared, and the protein concentration was determined using a bicinchoninic acid protein assay kit. An equal amount of protein was used in each experiment. The supernatants were transferred to new tubes and incubated with anti-Bax antibodies at a dilution of 1:200 at 4 °C for 4 h. The immunoprecipitated pellets were subsequently incubated with protein A-Sepharose, washed three times with lysis buffer, and separated on SDS-PAGE. After electrophoresis, the gels were processed for immunoblotting with anti-Bax (1:3000), anti-ubiquitin (1:1000) and anti-USP24 (1:3000) antibodies.

**Immunofluorescence Microscopic Analysis**

A549 cells were seeded onto glass slides overnight and fixed with 4% paraformaldehyde in phosphate-buffered saline (PBS) at 4 °C for 15 min. The cells were then rinsed with PBS two times and permeated with 1% Triton X-100 for 7 min. Next, the cells were pretreated with 1% bovine serum albumin (BSA) in PBS at room temperature for 60 min, incubated with antibodies at a dilution of 1:200 for 1 h, and treated with FITC-conjugated donkey anti-mouse immunoglobulin G (IgG) polyclonal antibodies and cyanine 5-conjugated donkey anti-rabbit IgG polyclonal antibodies (Jackson ImmunoResearch Laboratories, Inc.) at a dilution of 1:250 for 1 h. Finally, the cells were washed with PBS, mounted in 90% glycerol containing DAPI, and analyzed using an immunofluorescence microscope (Personal DV Applied Precision, Issaquah, WA) with deconvolution function (softWORX).

**cDNA Microarray**

A549 cells were infected with scramble or shUSP24 expressing lentivirus for 4 days, and total RNA was extracted by using TRIsure (Bioline, Taunton, MA, USA). Extracted RNA was analyzed by using microarray analysis (Phalanx Biotech, Hsinchu, Taiwan).

**Time-Lapse Study**

For time-lapse study and imaging, H1299 cells were transfected with GFP-USP24 and visualized under a fluorescent microscope (Olympus IX81-ZDC Zero Drift microscope). Phase contrast and green fluorescent protein (GFP) images were acquired every 10 min (at 5 sec exposure) for 14 hr. The cells were incubated in complete medium at 37°C and 5% CO_2_.

**Protein Stability Assay**

Cells were infected with scramble or shUSP24 shRNA expressing lentivirus for 4 days, and treated with 100 μg/ml cycloheximide (Sigma-Aldrich) to inhibit protein translation. Cells were resolved in sample buffer at indicated time, and protein stability was analyzed by western blot. Protein level was quantified by using Multi Gauge 3.0 software (Fujifilm, Japan).

**Xenograft Study**

The animal experiment was approved by the Institutional Animal Care and Use Committee at National Cheng Kung University. Female SCID mice were purchased from National Laboratory Animal Center in Taiwan. A549 luciferase expressing cells (A549-luc) were infected with scramble and shUSP24 lentivirus in a dose of 5 m.o.i. for 24 hours, and implanted into the back of severe combined immune-deficient (SCID) mice. After 3 weeks of implantation, tumor area was detected by non-invasion in vivo imaging system (IVIS) (PerkinElmer, Waltham, Massachusetts, USA). Tumors were resected 4 weeks after implantation and weighted.

**Two-Hybrid Screen**

The DNA fragments encoding a portion of human USP24 were generated by polymerase chain reaction and subsequently inserted into the pBTM116 vector to produce baits for yeast two-hybrid studies. The LexA-USP24 construct was used to screen against with human testis cDNA library (CLONTECH). Yeast two-hybrid screen was performed as described previously. L40 yeast strain was first transformed with pLexA-USP24 and followed by 200 mg of the testis cDNA library transformation. The library transformants were selected on medium lacking histidine, leucine, and tryptophan. His+ colonies were further tested for β-galactosidase activity using a colony lift filter assay. The plasmids from both His+ and X-gal+ colonies were isolated by the curing process of MC1066 bacterial strain and retransformed with LexA-USP24, or LexA-lamin to test the binding specificity. The library plasmids conferred that the USP24-specific interactions were then subjected to DNA sequence analysis.

**Nano-LC-MS/MS Analysis**

The dried hydrophilic interaction liquid chromatography fractions were reconstituted in 10 μl of buffer C (0.2% formic acid in deionized H_2_O) and analyzed by LTQ Orbitrap XL (San Jose, CA). Reverse phase nano-LC separation was performed on an Agilent 1200 series nanoflow system (Agilent Technologies, Santa Clara, CA). A total of 8 μl of sample from each hydrophilic interaction liquid chromatography fraction was loaded onto an Agilent Zorbax XDB C18 precolumn (inner diameter 0.3 × 5 mm, 5 μm), followed by separation using a C18 column (inner diameter 0.075 × 250 mm, 3 μm) from Micro Tech (Fontana). Buffer C was 0.1% folic acid, and buffer solution D was 0.1% folic acid in 98% acetonitrile. A linear gradient from 5 to 35% D over a 170-min period at a flow rate of 300 nl/min was applied. The peptides were analyzed in the positive ion mode by electrospray ionization (spray voltage = 1.8 kV). The mass spectrometer was operated in a data-dependent mode, in which one full scan was performed with m/z 300–2000 in the Orbitrap (resolution = 60,000 at m/z 400) using a rate of 30 ms/scan. The five most intense peaks for fragmentation with a normalized collision energy value of 35% in the LTQ were selected. A repeat duration of 30 s was applied to exclude the same m/z ions from the reselection for fragmentation. Peptide/protein identification was first performed with the Mascot search engine (available on the Matrix Science Web site).

**Mitochondrial Fractionation**

The cells were washed twice with phosphate-buffered saline (PBS) and scraped in PBS containing proteinase inhibitors. They were collected using a centrifuge at 1200 g for 5 min and then resuspended in 2 ml of MTE solution [270 mM D-mannitol, 10 mM Tris (pH 7.4), 0.1 mM EDTA]. Sonicate the cell suspension on ice three times for 10 sec each, separated by 10-sec rest intervals. Centrifuge the lysed cells for 10 min at 1400 g, and then collect 100 μl of supernatant from each tube as cytosol protein. Centrifuge decanted remaining supernatant for 10 min at 15000 g, and then resuspend the mitochondrial pellet in 0.8 ml of ice-cold 1x MTE plus proteinase inhibitors. Load slowly on top of mitochondrial sucrose gradient in a Beckman polyallomer ultracentrifuge tube for 22 min at 40000 g [gradient top: 1.6 ml of 1.0 M sucrose; gradient bottom: 1 ml of 1.7 M sucrose]. Collect mitochondrial fractions from the band at the interface of the 1.7 M and 1.0 M sucrose layers. Add 1.1 ml of ice-cold MTE, mix well and then centrifuge 10 min at 15000 g. Resuspend pellet with 1x sampling buffer as mitochondrial protein.

**Lentivirus-mediated gene knockdown**

USP24 shRNA (TRCN0000245778 and TRCN0000245779) was purchased from National RNAi Core Facility in Academia Sinica of Taiwan (Taipei, Taiwan), and shRNA-expressed lentiviruses were prepared by the RNAi Core of Research Center of Clinical Medicine, National Cheng Kung University Hospital (Tainan, Taiwan).

**GFP-USP24 Expression Plasmid Construction**

USP24 coding sequences was analyzed by NEBcutter V2.0 web site and two KpnI restriction enzymes sites and one BamHI restriction enzymes sites were found. USP24 gene was divided into four fragments and constructed separately according to the restriction enzymes sites, and BglII and BamHI restriction enzymes sites were added to the primer of first and last fragments. Primers used for construction are described in Supplementary Table 4. The four fragments of USP24 gene were fist ligated into yT&A vectors individually. Then the inserted USP24 fragments were removed from these four yT&A-USP24 constructs by restriction enzyme digestion and ligated into green fluorescent protein (GFP) vector, respectively, to form GFP-USP24 expression plasmid.

**Supplementary Figure Legends**

**Supplementary Figure S1.** EGF-inhibited USP24 expression induces cancer formation - A-B. EFGR^L858R^- (A) and Kras^G12D^- (B) induced lung cancer mice were treated with doxycycline for different time points, samples were collected from lung samples of mice, and then studied the cancer formation with IHC. C-E. Total RNA were extracted from primary lung cells (C), A431 cells with gefitinib treatment (D) and Kras^G12D^/EGFR^L858R^-induced mice (E) for RT-PCR, and then quantified the levels of USP24 mRNA after three independent experiments. F. USP24 was knocked down in A549 cells, and then cell morphology was observed. G. The cell numbers were evaluated in A549 cells with different doses of lenti-shUSP24 virus (a) and USP24 level shown here as an internal control (b). H. The cell numbers were evaluated in lung primary cells with USP24 knockdown (a) and USP24 level shown here as an internal control (b). I. The cell numbers were evaluated in A549 cells with or without USP24 knockdown (#2 clone:TRCN0000245778).

**Supplementary Figure S2**. USP24 induces apoptosis by stabilizing p300 and Bax - A. GFP only was transfected into H1299 cells and the cellular morphology was assessed using time-lapse fluorescence microscopy. B. Samples were harvested from GFP-USP24-over expressing and USP24-knockdown cells, and the Bax, caspase-3, USP24, EGFP-USP24, GFP and actin levels were examined by Western blotting with antibodies against the indicated proteins. The Bax, USP24 and GAPDH mRNA levels were evaluated by RT-PCR (a). The Bax level was quantified for the statistical analysis after three independent experiments (b). C. U2OS cells with USP24 knockdown were treated with cycloheximide and harvested the samples at the indicated time points for Western blotting with an anti-USP24 and anti-Bax antibodies (a). The level of Bax was quantified after three independent experiments (b). D. Cells with USP24 knockdown was treated with MG132, samples were harvested for Western blotting with antibodies against the indicated proteins. E. USP24 was knocked down with different dose of lenti-shUSP24 virus. Samples were harvested for Western blotting with antibodies against the indicated proteins. F. Several DNA sequences of USP24 including USP24, USP19, USP7, USP15 and USP22 were aligned for Cysteine Box. G. GFP, GFP-USP24 and GFP-USP24(C1698A) were expressed in cells individually. Cells were harvested for Western blotting with antibodies against ubiquitin, GFP and actin. H. GFP-USP24 or GFP-USP24(C1698A) was expressed in cells. Cells were harvested for Western blotting with antibodies against the indicated proteins. I. Various truncated Lex A-USP24 (a) and Gal AD-Ku70 were constructed for yeast two-hybrid assay. J. USP24 was knocked down in cells, and then cells were harvested for immunoprecipitation assay with anti-Ku70 (a) and anti-acetyl-lysine antibody (b). IP samples were analyzed by Western blotting with antibodies against the indicated proteins. K. USP24 was knocked down by #2 clone (TRCN0000245778) to study the level of Bax and p300.

**Supplementary Figure S3.** USP24 inhibits G1/S transition by increasing the E2F4 level - A. USP24 was knocked down and then total RNA was isolated for performing cDNA array. B. USP24 was knocked down in A549 (a) or U2OS (b) cells, and then cells were harvested for flow-cytometry assay. The ratio of G0/G1 and S-phases were quantified in U2OS cells (c). C. The binding motifs of E2F, TTTGGCG, within the promoters of CCNA2 and E2F1 were shown. D. USP24 was knocked down in A549 cells, and then samples were harvested for flow-cytometry assay. E. USP24 was knocked down in A549 cells, and then samples were harvested for Western blotting with antibodies against the indicated proteins (a). Data was quantified after three independent experiments (b).F. USP24 was knockdown in A549 cells, and then total RNA was isolated for RT-PCR to study the p130, TFDP1 and GAPDH mRNA levels (a). Data was quantified after three independent experiments (b). G. USP24 was knocked down by #2 clone (TRCN0000245778) to study the level of E2F4. H. USP24 was knocked down in A549 cells, and then samples were harvested for Western blotting with antibodies against the indicated proteins (a). Data was quantified after three independent experiments (b). I. USP24 was knocked down in U2OS cells, and cells after CHX treatment were harvested at different time points for Western blotting with antibodies against USP24, E2F4 and actin (a). Data was quantified after three independent experiments (b).

**Supplementary Figure S4.** A decrease in USP24 in mitosis is crucial for the metaphase-anaphase transition – A. USP24 was knockdown in cells, and then cells were harvested for Western blot with antibodies against indicated proteins. B. USP24 was knockdown in cells for flow-cytometry assay. C. A549 cells were treated with nocodazole release and then harvested the cells after different time points. Samples were analyzed by Western blot with antibodies against USP24, cyclin B1 and actin. D. GFP-USP24 was expressed in HeLa cells, and then cells were harvested for Western blot with antibodies against GFP and actin antibodies. E. HeLa cells treated with nocodazole and MG132 were harvested for Western blot with antibodies against indicated proteins. F. A549 cells treated with nocodazole were harvested for Western blot with antibodies against indicated proteins. G. HeLa cells treated with nocodazole or with nocodazole release were harvested at different time points. Samples were analyzed by Western blot with antibodies against indicated proteins. H. U2OS cells transfected with GFP-USP24 were fixed for immunofluorescence assay with antibodies against the indicated proteins, cdc20 (red), GFP-USP24 (green), DAPI (blue). I. HeLa cells treated with nocodazole were harvested for immunoprecipitation assay with anti-USP24 antibodies. IP samples were analyzed by Western blot with antibodies against indicated proteins. J. USP24 was knockdown in A549 cells and then cells were harvested at different time points after nocodazole release treatment. Samples were analyzed by Western blot with antibodies against GFP-USP24, cycling B1 and actin (a). The level of cyclin B1 was quantified (b). K. GFP-USP24 was overexpressed in HeLa cells, and then cells were fixed for immunofluorescence assay with antibodies against the indicated proteins, securin (red), GFP-USP24 (green), DAPI (blue). L. A549 (a) and HeLa cells (b) were treated with nocodazole. Samples were collected after nocodazole treatment (NZ) or nocodazole release (NZ-R). Samples were analyzed by Western blot with antibodies against indicated proteins. M. U2OS cells with USP24 knockdown (a) or GFP-USP24 overexpression (b) were treated with nocodazole. Samples were analyzed by Western blot with antibodies against the indicated proteins.

**Supplementary Figure S5.** Regulation of USP24 by phosphorylation increases the degradation of USP24 - A. Samples were harvested from HeLa cells that remained in interphase (I) and mitosis (M) for the immunoprecipitation assay with an anti-USP24 antibody. The IP samples were analyzed by Western blotting with antibodies against USP24, CDK1, cyclin B1, phospho-Ser/Thr and actin. B. Various mutations of USP24 phosphorylation residues were constructed and expressed in cells. Samples were analyzed by Western blotting with an anti-GFP antibody. C. Non-phospho-peptide (NP-Pep) and peptides with phosphorylation (P-Pep) at Ser1616, Ser2047, or Ser2604 were synthesized for dot blot analysis with anti-non-phospho-USP24 (NP-Ab) antibody, or anti-phospho-USP24 (S1616), anti-phospho-USP24 (S2047) or anti-phospho-USP24 (S2604) (P-Ab) antibodies. D. Phosphorylation antibodies of USP24 were performed in USP24 knockdown cells. E. GFP-USP24, GFP-USP24-S1616, GFP-USP24-S2047 or GFP-USP24-S2604 overexpressed in U2OS cells, and then samples were harvested for the immunoprecipitation assay with a GFP antibody. The IP samples were analyzed by Western blotting with indicated phospho-antibodies. F. Samples were harvested from A549 cells treated with PI3K inhibitor, and then analyzed by Western blotting with antibodies against USP24 phosphorylation at the indicated residues (a). The relative levels were quantified after three independent experiments (b).

**Supplementary Figure S6.** USP24 is correlated with its substrates in lung cancer mice and clinical lung cancer samples. The results shown here are in whole based upon data generated by the TCGA Research Network:  [http://cancergenome.nih.gov/. Lung adenocarcinoma mRNA](http://cancergenome.nih.gov/.%20Lung%20adenocarcinoma%20mRNA) sequencing data (N = 519) was downloaded and correlation between USP24, p300, Bax, securin, and E2F4 were analyzed.
